# Whole blood versus red cell concentrates for children with severe anaemia: a secondary analysis of the Transfusion and Treatment of African Children (TRACT) trial

**DOI:** 10.1016/S2214-109X(21)00565-9

**Published:** 2022-02-15

**Authors:** Elizabeth C George, Sophie Uyoga, Bridon M'baya, Dorothy Kyeyune Byabazair, Sarah Kiguli, Peter Olupot-Olupot, Robert O Opoka, George Chagaluka, Florence Alaroker, Thomas N Williams, Imelda Bates, Dora Mbanya, Diana M Gibb, A Sarah Walker, Kathryn Maitland, TRACT trial study group, Kathryn Maitland, Sarah A Walker, Elizabeth C George, Thomas N Williams, Diana M Gibb, Ayub Mpoya, Gary Frost, Kevin Walsh, Peter Olupot-Olupot, Julius Nteziyaremye, Cate Namayanja, Tonny Ssenyondo, George Passi, Rita Muhindo, George Masifa, Ruth Adong, Charles B Okalebo, Emmanuela Atimango, Nobert Thembo, George Odong, Godfrey Kiluli, Job Kapsindet, Sarah Kiguli, Robert O Opoka, Julianne Kayaga, Eva Nabawanuka, Eva Kadama, Cynthia Williams Mukisa, Charles Engoru, Florence Alaroker, Margaret Nakuya, Denis Amorut, Moses Olupot, Pius Onyas, Margaret Ariimi, Melda Itipe, Mary G Atim, Mary Abeno, Steven Okwi, Mary G Kulume, Grace Among, Dorreen E Achipa, Sophie Uyoga, Alex Macharia, Machpherson Mellewa, George Chagaluka, Neil Kennedy, Felistas Kumwenda, Tiferanji Fatch Sochera, Albert Malenga, Yamikani FG Chimalizeni, Benard Gushu, Tusekile Phiri, Amisa Chesale, Ndaona Mitole, Ellida Chokani, Annie Munthali, Michael Boele von Hensbroek, Annabelle South, Margaret J Thomason, David Baptiste, Roisin Connon, Leanne MacCabe, Abdul Ali, Kibibi Khamis, Macreen Madoola, Grace Abongo, Imelda Bates, Britta Urban, Robert Heydermann, Flavia Kyomuhendo, Sarah Nakalanzi, John Chabuka, Nkhafwire Mkandawire, Diana M Gibb, Felicity Fitzgerald, Jennifer A Evans, Elizabeth Molyneux, Irene Lubega, Jane Crawley, Peter Kazembe, Mike Murphy, Tim Peto, Jim Todd, Grace Mirembe, Philipa Musoka, Filemoni Tenu

**Affiliations:** a Medical Research Council Clinical Trials Unit, University College London, London, UK; b Kenya Medical Research Institute, Wellcome Trust Research Programme, Kilifi, Kenya; c Malawi Blood Transfusion Services, Blantyre, Malawi; d Uganda Blood Transfusion Services, Kampala, Uganda; e Department of Paediatrics and Child Health, School of Medicine, Makerere University, Uganda; f Busitema University Faculty of Health Sciences, Mbale Regional Referral Hospital, Mbale, Uganda; g Mbale Clinical Research Institute, Mbale, Uganda; h College of Medicine, Malawi-Liverpool-Wellcome Research Programme, Blantyre, Malawi; i Soroti Regional Referral Hospital, Soroti, Uganda; j Department of Infectious Disease, Institute of Global Health and Innovation, Imperial College London, London, UK; k Liverpool School of Tropical Medicine, Liverpool, UK; l Haematology and Transfusion Service, Centre Hospitalier et Universitaire, Yaounde, Cameroon

## Abstract

**Background:**

The TRACT trial established the timing of transfusion in children with uncomplicated anaemia (haemoglobin 4–6 g/dL) and the optimal volume (20 *vs* 30 mL/kg whole blood or 10 *vs* 15 mL/kg red cell concentrates) for transfusion in children admitted to hospital with severe anaemia (haemoglobin <6 g/dL) on day 28 mortality (primary endpoint). Because data on the safety of blood components are scarce, we conducted a secondary analysis to examine the safety and efficacy of different pack types (whole blood *vs* red cell concentrates) on clinical outcomes.

**Methods:**

This study is a secondary analysis of the TRACT trial data restricted to those who received an immediate transfusion (using whole blood or red cell concentrates). TRACT was an open-label, multicentre, factorial, randomised trial conducted in three hospitals in Uganda (Soroti, Mbale, and Mulago) and one hospital in Malawi (Blantyre). The trial enrolled children aged between 2 months and 12 years admitted to hospital with severe anaemia (haemoglobin <6 g/dL). The pack type used (supplied by blood banks) was based only on availability at the time. The outcomes were haemoglobin recovery at 8 h and 180 days, requirement for retransfusion, length of hospital stay, changes in heart and respiratory rates until day 180, and the main clinical endpoints (mortality until day 28 and day 180, and readmission until day 180), measured using multivariate regression models.

**Findings:**

Between Sept 17, 2014, and May 15, 2017, 3199 children with severe anaemia were enrolled into the TRACT trial. 3188 children were considered in our secondary analysis. The median age was 37 months (IQR 18–64). Whole blood was the first pack provided for 1632 (41%) of 3992 transfusions. Haemoglobin recovery at 8 h was significantly lower in those who received packed cells or settled cells than those who received whole blood, with a mean of 1·4 g/dL (95% CI –1·6 to –1·1) in children who received 30 mL/kg and –1·3 g/dL (–1·5 to –1·0) in those who received 20 mL/kg packed cells versus whole blood, and –1·5 g/dL (–1·7 to –1·3) in those who received 30 mL/kg and –1·0 g/dL (–1·2 to –0·9) in those who received 20 mL/kg settled cells versus whole blood (overall p<0·0001). Compared to whole blood, children who received blood as packed or settled cells in their first transfusion had higher odds of receiving a second transfusion (odds ratio 2·32 [95% CI 1·30 to 4·12] for packed cells and 2·97 [2·18 to 4·05] for settled cells; p<0·001) and longer hospital stays (hazard ratio 0·94 [95% CI 0·81 to 1·10] for packed cells and 0·86 [0·79 to 0·94] for settled cells; p=0·0024). There was no association between the type of blood supplied for the first transfusion and mortality at 28 days or 180 days, or readmission to hospital for any cause. 823 (26%) of 3188 children presented with severe tachycardia and 2077 (65%) with tachypnoea, but these complications resolved over time. No child developed features of confirmed cardiopulmonary overload.

**Interpretation:**

Our study suggests that the use of packed or settled cells rather than whole blood leads to additional transfusions, increasing the use of a scarce resource in most of sub-Saharan Africa. These findings have substantial cost implications for blood transfusion and health services. Nevertheless, a clinical trial comparing whole blood transfusion with red cell concentrates might be needed to inform policy makers.

**Funding:**

UK Medical Research Council (MRC) and the Department for International Development.

**Translation:**

For the French translation of the abstract see Supplementary Materials section.

## Introduction

Most transfusions in sub-Saharan Africa are done using whole blood.^1^ Reflecting international practice, blood transfusion services across sub-Saharan Africa are increasingly moving towards a wider provision of red cell concentrates, despite clear differences in the patient populations requiring transfusion.^2^ Although red cell concentrates are more costly and time-consuming to prepare, these changes have mostly been justified on the grounds of maximising the use of a single blood donation. Data on the requirements for blood components are scarce for most countries in Africa. Studies published on this topic tend to be biased towards blood transfusion services,^3^, ^4^ and might not represent the needs of less-resourced hospital blood banks. Generally, requirements differ from those in high-income countries, in which oncology and surgery use most of the transfusion components; whereas in sub-Saharan Africa demand is predominantly centred around the high incidence of acute paediatric severe anaemia (mainly due to nutritional and infectious causes [malaria, sepsis, and helminths] and sickle cell disease), haemorrhagic complications of pregnancy, and trauma.^5^ Transfusions required by these patient groups are mainly for emergency management in cases where whole blood needs to be replaced. Evidence supporting the superiority of red cell components (packed cells) over whole blood in children and pregnant women, the most common recipients in sub-Saharan Africa, is weak.^6^ The use of whole blood for African children admitted to hospital with severe anaemia does not lead to fluid overload events^7^ and was shown to be safe, but the sample size in most studies is too small to provide reliable information for guideline recommendations. With respect to international guidelines for paediatric transfusion in children who are critically ill, in 2018, the international Transfusion and Anemia Expertise Initiative developed consensus recommendations for red cell transfusion.^8^ The guideline group noted the general paucity of published paediatric data to justify their recommendations. Moreover, there were no specific recommendations on the use of whole blood versus red cell concentrates.

**Research in context**

**Evidence before the study**

In sub-Saharan Africa, blood transfusion services are moving towards provision of red cell concentrates rather than using whole blood for transfusion, reflecting practice in high-income countries. These changes have mainly been justified on the grounds of maximising the use of a single blood donation, despite red cell concentrates being more costly and time-consuming to prepare than whole blood. We searched PubMed and Google Scholar for original articles and systematic reviews published between database inception and June 10, 2021, involving treatment recommendations for transfusion for paediatric severe anaemia in Africa, using the terms ”transfusion”, “severe anaemia”, “child”, and “guideline” with no language restrictions. A systematic review on transfusion practice in sub-Saharan Africa in 2019 evaluated the recommendations and guidelines supporting whole blood and packed red cell transfusions for pregnancy-related indications and paediatric anaemia. From 15 countries in sub-Saharan Africa, 32 guidelines in English were identified. Seven guidelines justified the use of red cell concentrates rather than whole blood, mostly on the basis of safety (whole blood was considered to increase the risk of volume-related complications). None of these guidelines cited research findings to support their recommendations. In 2018, the international Transfusion and Anemia Expertise Initiative developed consensus recommendations on red blood cell transfusion in children who were critically ill. Notably, one major challenge for developing and justifying their recommendations was the paucity of published paediatric data. No specific recommendations were made on the use of whole blood versus red cell concentrates. Before our TRACT trial, evidence to support the introduction of red cell concentrates for paediatric transfusion in Africa was scarce. Additionally, no clinical trials have compared whole blood and packed red cell transfusion with patient outcomes.

The multicentre, factorial, TRACT trial was designed to establish evidence-based transfusion practice. The trial investigated immediate transfusion versus no transfusion (standard of care) in children with uncomplicated severe anaemia (haemoglobin 4–6 g/dL) and whether a higher volume of whole blood (30 mL/kg or red cell concentrate equivalent) in children with haemoglobin lower than 6 g/dL would improve outcomes compared with the standard volume (20 mL/kg whole blood or red cell concentrate equivalent). Outcomes included mortality until day 28 (primary outcome) and day 180, readmissions, and relapse of severe anaemia. The trial showed that non-immediate transfusion in children with uncomplicated severe anaemia is safe; provided that children are monitored (for the development of severity). The TRACT trial also showed that higher volumes of blood transfusions reduced the number of deaths in children without fever (hazard ratio [HR] 0·43, 95% CI 0·27–0·69). Whereas in children admitted to hospital with fever (>37·5°C) who received 30 mL/kg whole blood equivalent, the risk of mortality almost doubled (HR 1·91, 95% CI 1·04–3·49) compared with those who received a lower volume of 20 mL/kg. Transfusion with whole blood and red cell concentrates did not affect the primary or safety endpoints.

**Added value of this research**

The secondary analysis of the TRACT trial examined the optimal pack type in 3992 transfusions, of which 1632 (41%) were whole blood transfusions and 2360 (49%) were red cell concentrate transfusions (issued by the blood bank but not prespecified on the request form). The analysis of haemoglobin recovery by pack type at 8 h showed superior recovery in children initially receiving whole blood (p<0·0001) versus red cell concentrates. Children who received an initial red cell concentrate transfusion had more second transfusion episodes and longer hospital stay than children who received whole blood initially. The pack type supplied for the initial transfusion did not predict any other clinical outcomes, including mortality at 28 or 180 days, or hospital readmissions. Transfusion-related adverse events, such as volume overload, were rare and unrelated to pack type. Although not randomised, observational analyses of trial data indicate that children who received red cell concentrates as the first transfusion had poorer haematological correction, additional transfusions (which increases infection risk because of increased donor exposure and further depletes scarce blood supplies), and longer hospital stays without any safety benefit than children who received whole blood.

**Implications of all the available evidence**

Providing whole blood for paediatric transfusion, which accounts for a large proportion of transfusions in sub-Saharan Africa, could result in substantial cost and resource savings for blood transfusion and health services.

In 2015, a multicentre, factorial, open-label TRACT trial was conducted to establish evidence-based transfusion and treatment strategies with the aim of improving early and long-term mortality and readmission to hospital.^9^ The trial was registered (ISRCTN84086586) in Feb 11, 2013. The trial investigated immediate transfusion versus no transfusion (standard of care) in 1565 children with uncomplicated severe anaemia (haemoglobin 4–6 g/dL) and whether a higher volume of whole blood (30 mL/kg or red cell concentrate equivalent) with haemoglobin lower than 6 g/dL would improve outcomes compared with the standard volume (20 mL/kg whole blood or red cell concentrate equivalent) in 3196 children. The outcomes were mortality until day 28 (primary outcome) and day 180, readmissions, and safety (appendix 2 p 2). By day 28, the number of deaths reduced in children without fever who received 30 mL/kg whole blood compared with those who received 20 mL/kg (hazard ratio [HR] 0·43, 95% CI 0·27–0·69). Whereas in children who had fever (>37·5°C) and received 30 mL/kg of whole blood the risk of mortality almost doubled at 28 days compared with those who received 20 mL/kg (HR 1·91, 95% CI 1·04–3·49; heterogeneity p<0·0001).^10^ Serious adverse events (mostly readmissions) occurred in 431 (27%) of 1598 children in the higher volume group versus 416 (26%) in the lower volume group: allergic reactions occurred in 25 (2%) versus 20 (1%) children (p=0·55; no deaths), suspected pulmonary or cardiovascular serious adverse events occurred in 2 (<1%) versus 3 children (<1%; p=1·00; two deaths), and a neurological serious adverse event (grade 3) occurred in one child in the higher volume group (appendix 2 p 4). The additional transfusion randomisation (n=1565) comparing immediate transfusion with no immediate transfusion (control) was reported separately.^11^

Given the paucity of data on the merits of different pack types on clinical outcomes in the management of children admitted to hospital with severe anaemia, the main objective of this secondary analysis was to examine the safety and efficacy of different pack types (whole blood *vs* red cell concentrates) on haematological correction, retransfusion, mortality, and readmission to hospital.

## Methods

### Study design and participants

This study is a secondary analysis of the TRACT trial^10^ data restricted to those who received an immediate transfusion (using whole blood or red cell concentrates). TRACT was an open-label, multicentre, factorial, randomised trial conducted in three hospitals in Uganda (Soroti, Mbale, and Mulago) and one hospital in Malawi (Blantyre). The trial enrolled children aged between 2 months and 12 years admitted to hospital with severe anaemia (haemoglobin <6 g/dL). A complete list of the inclusion and exclusion criteria is shown in the appendix 2 (p 1). Written informed consent was obtained from parents or guardians. When written consent could not be obtained (in cases where the child was at imminent risk of death or parent was not able to provide informed consent), ethics committees approved verbal parental or guardian assent with delayed written informed consent.^12^ The ethics committees of Imperial College London (London, UK; ICREC_13–1-11), Makerere University (Kampala, Uganda; SOMREC 2013–050), and College of Medicine (Blantyre, Malawi; COMREC P.03/13/1365) approved the trial protocol,^9^ from which we conducted this secondary analysis.

### Procedures

Details on the management and conduct of this study have been published previously.^10^ Briefly, a structured clinical case report form and baseline investigations were completed at admission to hospital. Bedside observations were performed at admission and every 30 min for the first 2 h, then at 4, 8, 16, 24, and 48 h after the start of the first transfusion. Haemoglobin was assessed using HemoCue Hb 301 system (HemoCue, Angelholm, Sweden) every 8 h in the first 24 h, then at 48 h, or if triggered by clinical deterioration.^13^ Patients were actively monitored for serious adverse events, particularly suspected cardiac or pulmonary overload or transfusion-related events, following modified guidelines recommended by the UK Serious Hazards of Transfusion initiative.^14^ Post-discharge, children were clinically assessed and haemoglobin was measured at 28, 90, and 180 days.

In the trial all blood products were supplied free of charge to patients and hospitals. Red cell concentrates were prepared using gravity (settled cells) or by centrifugation (packed cells).^15^ The pack type used (supplied by blood banks) was based only on availability at the time and not on clinician preference, patient need, or the trial protocol (appendix 2 pp 1–2).

Whole blood was transfused over 3–4 h and packed or settled cells were transfused over 2–3 h. Additional transfusions were permitted for new or persistent haemoglobin concentrations (<4 g/dL) or severity features (development of respiratory distress or impaired consciousness) with haemoglobin 4–6 g/dL, defined as clinical deterioration. At each clinical assessment, clinicians examined children for de novo features of severity, reductions in haemoglobin (requiring an additional transfusion), and actively observed any suspected transfusion-related adverse events (febrile reactions), transfusion-related acute lung injury, and transfusion-associated circulatory overload.^9^

### Outcomes

The outcomes were haemoglobin recovery at 8 h and 180 days, requirement for retransfusion, length of stay, changes in heart and respiratory rates until day 180, and the main clinical endpoints (mortality until day 28 and day 180, and readmission until day 180), measured in the intention-to-treat population.

### Statistical analysis

Baseline characteristics were summarised and children's haemoglobin concentrations (mean and 95% CI) were plotted over time and stratified by blood pack type (first pack from the first transfusion). The effect of blood pack type on different clinical outcomes was measured using multivariate regression models. These models were either previously published (for mortality and readmissions; appendix 2 pp 6–7, 8–11)^10^, ^16^ or built using backwards elimination with a threshold of p<0·1 and candidate covariates to enable adjustment for confounders, which were identified using clinical input, previous knowledge, reviewing literature, and checking for non-linearity of continuous variables with the Stata (version 16.1) multivariable fractional polynomial function (α=0·05). All models were adjusted for site, if not already included in model. Cox regression was used for mortality models and Schoenfeld residuals were tested and checked graphically for non-proportionality. Logistic regression was used to analyse children receiving a second transfusion during the initial hospital admission. Haemoglobin recovery at 8 h and 180 days was examined with normal linear regression, adjusted for baseline haemoglobin. Time to discharge was modelled using competing risks with the competing event defined as death during initial admission and a subhazard ratio of less than 1 indicating longer time to discharge; for time to readmission models, the competing event was defined as death post-discharge. Competing risk methods estimated the probability of an event (analogous to Kaplan-Meier) using cumulative incidence functions and the effect of each adjusted variable on the subdistribution hazard corresponding to the cumulative incidence function.^17^ Blood pack type was then added into these models, if not already retained, to examine whether the pack type was independently predictive of the outcome. Possible interactions in outcome models for blood pack type and randomisation group were considered before analyses and tested using a likelihood ratio test or Wald test and p values, checked graphically from the ten models, and compared with Benjamini-Hochberg critical values to adjust for multiple testing.^18^

The p values from heterogeneity tests (likelihood ratio test or Wald test on two df) on the ten models were ranked and compared with critical values from the Benjamini-Hochberg method to account for multiple testing and strong evidence was found only in the model for change in haemoglobin at 8 h (p=0·0002; critical value p=0·0050), and thus, results were presented separately by volume randomisation for this model. All other models had p values higher than their corresponding critical value and thus effect estimates were presented overall.

For models, we used complete case analysis because the missing data was less than 5% for baseline values. Absolute numbers and outcome events in children receiving different blood pack types were summarised with unadjusted effect estimates (appendix 2 pp 4–5). Respiratory rate and heart rate from randomisation to 48 h post-transfusion were modelled using change from baseline as the outcome in a normal generalised estimating equation model, adjusting for baseline values (appendix 2 pp 16–17).^19^ Sensitivity analyses were carried out with data restricted to Soroti and Mbale (Uganda) for comparison of whole blood versus settled cells and restricted to Mulago (Uganda) and Blantyre (Malawi) for comparison of whole blood versus packed cells. Analyses were also carried out for malaria and HIV subgroups (appendix 2 pp 17–18). Stata (version 16.1) was used for all statistical analyses.

### Role of the funding source

The funder of the study had no role in study design, data collection, data analysis, data interpretation, or writing of the report.

## Results

Between Sept 17, 2014, and May 15, 2017, 3199 children with severe anaemia were enrolled into the TRACT trial. Consent was declined by three participants post-randomisation (appendix 2 p 4) and 3196 children (1598 in each group [30 mL/kg versus 20 mL/kg whole blood equivalent groups]) were included in further analysis. 2418 (76%) participants had one or more severity feature (impaired consciousness, respiratory distress, haemoglobinurea, profound anaemia, or reported sickle cell disease), and 778 (24%) had uncomplicated severe anaemia. Seven children died before the transfusion and one had no form to confirm transfusion; thus, 3188 children were considered in our secondary analysis. 1338 (42%) of 3188 had a haemoglobin of less than 4 g/dL and 883 (28%) of 3160 had sickle cell disease confirmed by genotyping.

Baseline characteristics are shown in table 1. The median age was 37 months (IQR 18–64). 2045 (64%) of 3188 children had malaria, 589 (18%) had haemoglobinuria, and 1338 (42%) had a haemoglobin of less than 4 g/dL at screening. Although statistically significant due to the trial size, differences in baseline characteristics between blood pack types were small. Soroti and Mbale hospitals predominantly used whole blood and settled cells, the hospital in Blantyre predominantly used whole blood and packed cells, and the one in Mulago used all three pack types.

**Table 1:** Baseline characteristics of children included in this analysis

		**Type of first blood pack in initial transfusion**				p value*
		Whole blood pack (n=1404)	Red cell concentrates pack (packed cells; n=692)	Red cell concentrates pack (settled cells; n=1092)	Total (n=3188)	
Age						
	Median, months	39 (22–66)	37 (16–70)	33 (16–61)	37 (18–64)	0·0015
	Mean, months	47 (33)	47 (36)	42 (32)	45 (33)	0·0024
Sex						
	Male	803 (57%)	395 (57%)	612 (56%)	1810 (57%)	0·83
	Female	601 (43%)	297 (43%)	480 (44%)	1378 (43%)	..
Median haemoglobin, g/dL		4·2 (3·4–5·2)	4·3 (3·4–5·3)	4·2 (3·3–5·1)	4·2 (3·4–5·2)	0·46
Haemoglobin, <4 g/dL		580 (41%)	288 (42%)	470 (43%)	1338 (42%)	0·67
Mean temperature, °C		37·4 (1·0)	37·4 (1·0)	37·4 (0·9)	37·4 (1·0)	0·34
Mean heart rate, bpm		146 (21)	145 (23)	148 (23)	146 (23)	0·0075
Severe tachycardia†		351 (25%)	166 (24%)	306 (28%)	823 (26%)	..
Median oxygen saturation		98% (95–99)	97% (95–98)	97% (95–99)	97% (95–99)	0·0007
Mean respiratory rate, breaths per min		42 (12)	45 (13)	44 (14)	44 (13)	0·0001
Tachypnoea‡		878 (63%)	495 (72%)	704 (64%)	2077 (65%)	..
HIV status						
	Positive	47 (4%)	35 (5%)	16 (2%)	98 (3%)	<0·0001
	Negative	1280 (96%)	636 (95%)	1026 (98%)	2942 (97%)	..
Malaria status						
	Positive	938 (67%)	375 (54%)	732 (67%)	2045 (64%)	<0·0001
	Negative	463 (33%)	316 (46%)	358 (33%)	1137 (36%)	..
Blood culture						
	Positive	39 (4%)	24 (4%)	29 (3%)	92 (3%)	0·70
	Negative	1071 (96%)	634 (96%)	949 (97%)	2654 (97%)	..
Median C-reactive protein, mg/dL		63·7 (24·5–112·0)	56·0 (18·5–126·0)	62·4 (25·9–113·0)	62·0 (23·8–114·4)	0·22
Median lactate, mmol/L		2·9 (2·0–4·9)	3·0 (2·1–4·4)	2·7 (1·7–4·5)	2·9 (1·9–4·6)	0·044
Mean glucose, mmol/L		5·8 (1·5)	5·4 (1·3)	5·7 (1·6)	5·7 (1·6)	<0·0001
Impaired consciousness§						
	Yes	332 (24%)	216 (31%)	204 (19%)	752 (24%)	<0·0001
	No	1072 (76%)	476 (69%)	888 (81%)	2436 (76%)	..
Haemoglobinuria¶						
	Yes	278 (20%)	97 (14%)	214 (20%)	589 (18%)	0·0029
	No	1126 (80%)	595 (86%)	878 (80%)	2599 (82%)	..
Sickle cell genotype						
	AA	1035 (74%)	434 (63%)	713 (65%)	2182 (68%)	<0·0001
	AS	42 (3%)	22 (3%)	31 (3%)	95 (3%)	..
	SS unknown	153 (11%)	148 (21%)	153 (14%)	454 (14%)	..
	SS known	164 (12%)	72 (10%)	193 (18%)	429 (13%)	..
Patient blood group						
	A	362 (26%)	171 (25%)	295 (27%)	828 (26%)	0·0104
	B	345 (25%)	152 (22%)	288 (26%)	785 (25%)	..
	AB	83 (6%)	25 (4%)	63 (6%)	171 (5%)	..
	O	614 (44%)	343 (50%)	446 (41%)	1403 (44%)	..
Mean pack haemoglobin, g/dL		14·8 (3·3)	19·5 (2·5)	16·9 (2·8)	16·6 (3·5)	<0·0001
Mean pack haematocrit		47·1 (13·8%)	60·4 (8·4%)	53·4 (11·2%)	52·0 (13·0%)	<0·0001
Mean pack storage age, days		12 (9)	14 (9)	14 (8)	13 (9)	0·0003
Site						
	Blantyre, Malawi	318 (23%)	77 (11%)	0	395 (12%)	<0·0001
	Mulago, Uganda	70 (5%)	602 (87%)	60 (5%)	732 (23%)	..
	Soroti, Uganda	616 (44%)	0	244 (22%)	860 (27%)	..
	Mbale, Uganda	400 (28%)	13 (2%)	788 (72%)	1201 (38%)	..

Data are shown as median (IQR), mean (SD), and n (%). bpm=beats per minute.

* p value calculated from a χ^2^ test for categorical variables, a K-sample equality-of-medians test for continuous variables comparing medians, and a one-way ANOVA for comparing means.

† Severe tachycardia defined as heart rate higher than 180 bpm (age <1 year), 160 bpm (age 1–4 years), or 140 bpm (age ≥5 years).

‡ Tachypnoea defined as respiratory rate of 50 breaths per min or higher (age <1 year), 40 breaths per min or higher (age 1–4 years), or 30 breaths per min or higher (age ≥5 years).

§ Child is prostrate (inability to sit upright at age >8 months or to breastfeed at age ≤8 months) or in a coma (inability to localise a painful stimulus).

¶ Red or brown urine.

In the TRACT trial, 3992 transfusions were started in 3188 children during primary admission to hospital. 2692 (84%) children received one transfusion, 349 (11%) received two transfusions, and 147 (5%) received three or more transfusions during initial admission. Whole blood was the first pack provided for 1632 (41%) of 3992 transfusions, of which 1101 (67%) of 1632 were adult-size packs and 531 (33%) were from transfer packs (pedipacks). 844 (36%) of 2360 red cell concentrate transfusions were packed cells whereas 1516 (64%) were settled cells. Adherence to volumes directed by the protocol (20 mL/kg or 30 mL/kg for whole blood and 10 mL/kg or 15 mL/kg for packed or settled cells) for initial transfusions was within SD 1·5 mL of the expected volumes in 681 (98%) of 692 red cell concentrate transfusions for packed cells and 1029 (94%) of 1092 for settled cells, and within SD 3 mL of the expected volumes in 1367 (97%) of 1404 for whole blood transfusions.

486 children required two or more packs in their initial transfusion. 109 (22%) of 486 had two or more different pack types and one had missing information on the second pack type. 78 (72%) of 109 children received a different type of whole blood pack (ie, transfer instead of direct or vice versa), and four received a different type of red cell concentrate (ie, packed cell instead of settled cell or vice versa). 27 (25%) of 109 children received whole blood and red cell concentrate. Four received both red cell concentrate types (packed and settled cells).

The whole blood packs had a median storage age of 10·0 days (IQR 5·0–18·0) compared with 12·0 days (7·0–20·0) for packed cells and 13·0 days (7·0–19·0) for settled cells. As expected, the haemoglobin concentrations in packed cells and settled cells were higher than those in whole blood, with a similar finding in the haematocrits (table 2).

**Table 2:** Characteristics of the packs used in transfusions

	**Expected values for whole blood**	**Whole blood (n=1994 packs)**	**Expected values for red cell concentrates**	**Red cell concentrates (packed cells by centrifugation; n=968 packs)**	**Red cell concentrates (settled cells by gravity; n=1589 packs)**	**Overall (n=4551 packs)**
Pack haemoglobin, g/dL	>12·0	13·9 (12·6–16·0)	15·0–20·0	19·5 (17·7–21·1)	16·9 (15·0–18·7)	16·3 (13·8–19·0)
Pack haematocrit, %	35·0–45·0%	42·1% (38·0–52·0)	50·0–70·0%	60·0% (55·3–65·0)	52·1% (45·4–60·0)	50·9% (42·0–60·0)
Pack storage age, days	<36·0	10·0 (5·0–18·0)	<43·0	12·0 (7·0–20·0)	13·0 (7·0–19·0)	12·0 (6·0–19·0)

Data are shown as median (IQR), unless stated otherwise.

**Figure 1:**
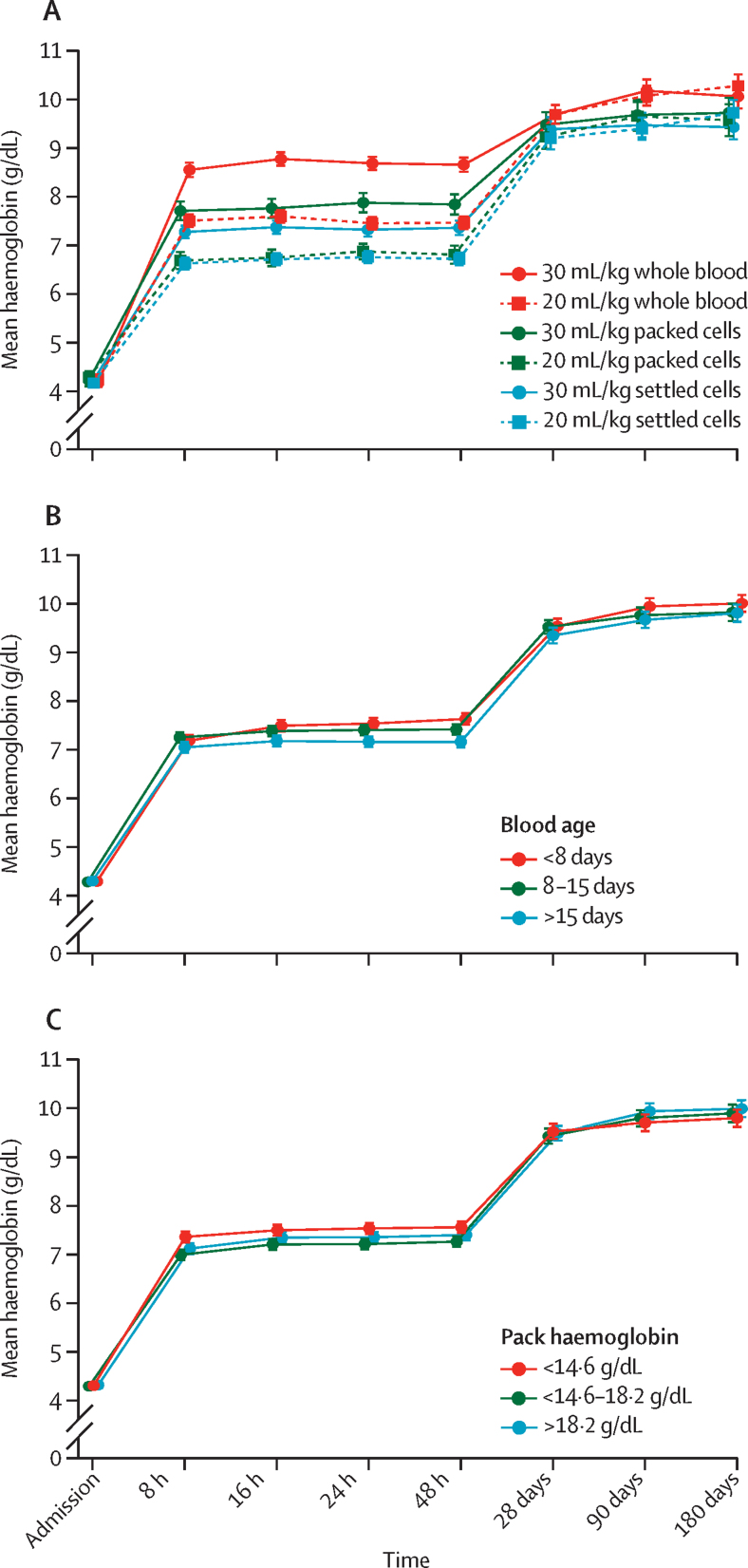
Haemoglobin recovery over time by blood pack characteristics Bars indicate 95% CIs. Haemoglobin recovery over time by type of first blood pack in first transfusion (A); by age of first blood pack in first transfusion (B); and by haemoglobin of first blood pack (C).

Children's mean haemoglobin recovery was greatest in the first 8 h following haemoglobin at admission (at time of random allocation) and stabilised before increasing further between 48 h and 28 days (figure 1A). Haemoglobin recovery at 8 h was significantly lower in those who received packed cells or settled cells than those who received whole blood, with a mean of 1·4 g/dL (95% CI –1·6 to –1·1) in children who received 30 mL/kg and –1·3 g/dL (–1·5 to –1·0) in those who received 20 mL/kg packed cells versus whole blood, and –1·5 g/dL (–1·7 to –1·3) in those who received 30 mL/kg and –1·0 g/dL (–1·2 to –0·9) in those who received 20 mL/kg settled cells versus whole blood (overall p<0·0001; p=0·0002 for heterogeneity between volumes; table 3; appendix 2 p 13). Haemoglobin recovery was slightly lower after transfusion with older blood (age >15 days) and blood packs with lower haemoglobin concentrations (figure 1B, C). There was no significant difference in haemoglobin recovery at 180 days for children receiving packed or settled cells compared with those receiving whole blood (table 3; appendix 2 p 14).

**Table 3:** The effect of each blood pack type supplied for transfusion

			**Adjusted estimate (95% CI) for packed cells versus whole blood packs**	**Adjusted estimate (95% CI) for settled cells versus whole blood packs**	**Overall Wald test p value**
**Time to event analyses (hazard ratio)**					
28-day mortality*			0·99 (0·48 to 2·04)	1·12 (0·64 to 1·95)	0·92
180-day mortality*			1·11 (0·66 to 1·85)	1·05 (0·75 to 1·46)	0·91
	Readmissions				
		All cause†	1·05 (0·66 to 1·65)	0·85 (0·68 to 1·06)	0·30
		Anaemia†	1·49 (0·87 to 2·57)	0·90 (0·69 to 1·16)	0·18
		Haemoglobinuria†	0·61 (0·12 to 3·02)	1·08 (0·70 to 1·67)	0·76
		Malaria†	0·59 (0·25 to 1·39)	0·70 (0·47 to 1·02)	0·13
	Time to discharge from hospital†		0·94 (0·81 to 1·10)	0·86 (0·79 to 0·94)	0·0024
**Mean change in haemoglobin analyses**					
8 h haemoglobin, g/dL (20 mL/kg)‡			−1·3 (−1·5 to −1·0)	−1·1 (−1·2 to −0·9)	<0·0001
8 h haemoglobin, g/dL (30 mL/kg)‡			−1·4 (−1·6 to −1·1)	−1·5 (−1·7 to −1·3)	<0·0001
180-day haemoglobin,‡ g/dL			0·0 (−0·4 to 0·4)	0·1 (−0·1 to 0·2)	0·57
**Second transfusions (odds ratio)**					
Odds of second transfusion§			2·32 (1·30 to 4·12)	2·97 (2·18 to 4·05)	<0·0001

All models were adjusted for site and each model was checked for heterogeneity of effect by volume randomisation.

* Analysed using Cox regression models.

† Competing risks regression models. For readmission, the competing event was death post-discharge and for time to discharge, the competing event was death during initial admission.

‡ Linear regression models.

§ Logistic regression model.

Compared to whole blood, children who received blood as packed or settled cells in their first transfusion had higher odds of receiving a second transfusion (odds ratio [OR] 2·32 [95% CI 1·30 to 4·12] for packed cells and 2·97 [2·18 to 4·05] for settled cells; p<0·001) and longer hospital stays (HR 0·94 [95% CI 0·81 to 1·10] for packed cells and 0·86 [0·79 to 0·94] for settled cells; p=0·0024; table 3; figure 1E; appendix 2 pp 12, 15). There was no association between the type of blood supplied for the first transfusion and the main clinical endpoints, including mortality at 28 days or 180 days, or readmission to hospital for any cause (table 3; appendix 2 p 19). There was no evidence of non-proportionality in the effect of pack type for mortality outcomes (Schoenfeld p>0·05).

**Figure 2:**
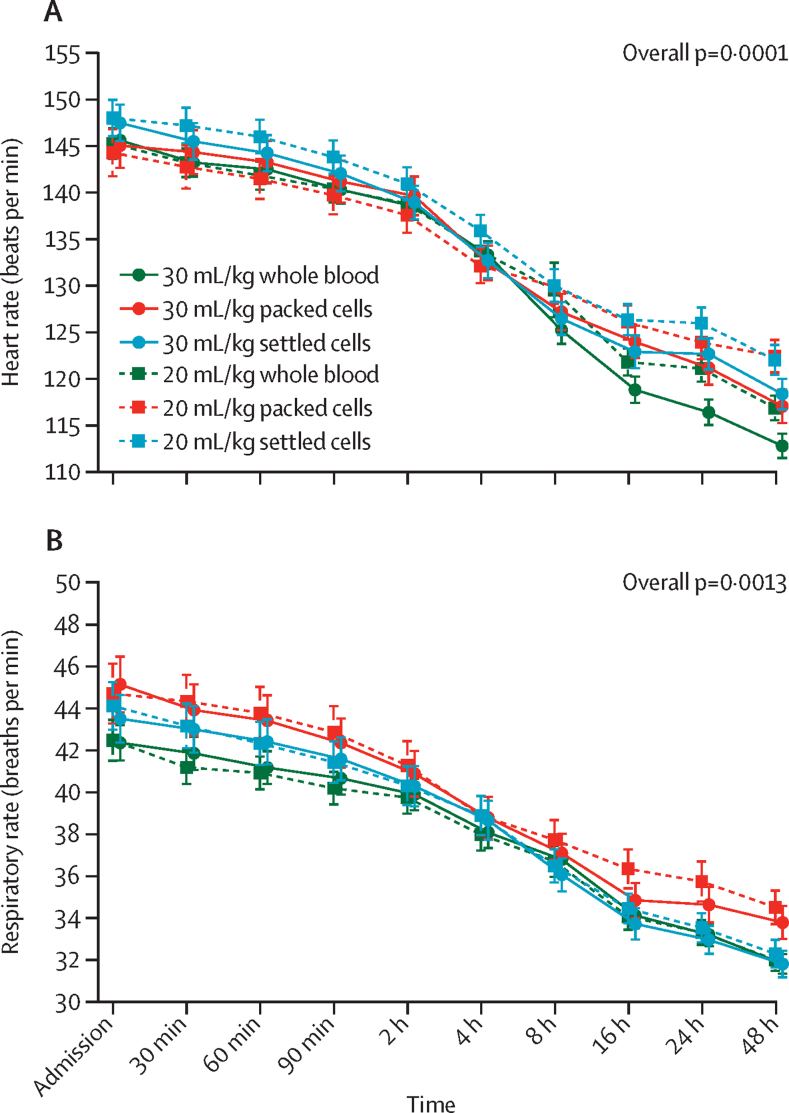
Heart rate and respiratory rate over time Bars indicate 95% CIs. Heart rate (A) and respiratory rate (B) over time by first blood pack type in first transfusion and by 20 mL/kg versus 30 mL/kg.

823 (26%) of 3188 children presented with severe tachycardia and 2077 (65%) with tachypnoea, but these complications resolved over time (figure 2; appendix 2 p 20). Heart rates decreased quicker in those receiving whole blood (overall p=0·0001), and respiratory rates (overall p=0·0013) decreased quicker in those receiving whole blood or settled cells (figure 2). Differences for heart rate at 48 h were mean 4·6 beats per min (bpm; 95% CI 0·3 to 8·8) in children who received 20 mL/kg whole blood and 3·1 bpm (1·1 to 5·0) in those who received 30 mL/kg packed cells and 4·0 bpm (0·1 to 7·9) in those who received 20 mL/kg whole blood and 4·8 bpm (3·1 to 6·5) in those who received 30 mL/kg settled cells. Differences for respiratory rate at 48 h were mean 1·5 (95% CI –0·4 to 3·4) breaths per min in children who received 20 mL/kg whole blood and 0·9 (–0·02 to 1·7) in those who received 30 mL/kg packed cells and 0·1 (–1·6 to 1·8) breaths per min in those who received 20 mL/kg whole blood and 0·01 (–0·7 to 0·7) in those who received 30 mL/kg settled cells. Results for malaria and HIV subgroups were similar to the analyses on all children (appendix 2 pp 17–18). Furthermore, only five episodes of suspected possible transfusion-related lung injury or cardiac overload were reported in the trial. Four of these episodes occurred in children receiving packed cells and one in a child receiving whole blood.

## Discussion

The multicentre TRACT trial investigated whether an initial transfusion with either whole blood or red cell concentrates (packed or settled cells) had an effect on clinical outcomes in children admitted to hospital with severe anaemia. We found that haematological correction at 8 h was substantially better in children who received whole blood than in those transfused with packed or settled cells, regardless of the volume of blood transfused (20 or 30 mL/kg). Children receiving red cell concentrates as their initial transfusion had a higher number of additional transfusions and longer hospital stay. There was no evidence that the type of blood was associated with mortality or readmissions.

These findings have important implications for blood transfusion services in Africa that are promoting the use of blood components, particularly given the additional staff and equipment requirements and consequently overall cost of preparing red cell concentrates. The Blood Transfusion Safety Programme for WHO recommends that blood transfusions collected might be used more effectively if they are separated into components (red cell concentrates, fresh frozen plasma, cryoprecipitates, and platelet concentrates), to be used by more than one patient. Moreover, the Global Database on Blood Safety uses the proportion of components prepared per unit of whole blood collected as a general indicator of productivity for blood transfusion services. A 2016, report^1^ indicated that 36% (15 of 41) of African countries contributing data to the audit had less than 25% of whole blood donations separated into components. Our findings indicate that achieving exclusive component preparation will have a negative effect on paediatric services, one of the largest users of transfused blood.^2^, ^20^

Potential volume overload is another reason for recommending red cell concentrates.^5^ In our study, 42% of children had haemoglobin of less than 4 g/dL and 27% had sickle cell disease. These groups might be at risk of volume overload or transfusion reactions, or both, since children with profound anaemia are considered at higher risk of heart failure and many children with sickle cell disease have had multiple previous transfusions.^11^, ^21^ However, we found no evidence of overload in children receiving whole blood compared with red cell concentrates, or with a larger volume of blood received, irrespective of the severity of anaemia or underlying sickle cell disease.^10^, ^22^ Most transfusion-related lung injuries or cardiac overloads were deemed by the endpoint review committee (who were unaware of treatment assignment) to be unrelated to the intervention.^10^ In the TRACT trial, no child received a diuretic or heart failure medication, and although there were differences between pack types in heart rate and respiratory rate at 48 h, these were too small to be clinically meaningful.

The major limitation of this study is that the trial data used for the subanalysis did not directly compare the use of whole blood with packed or settled cells using a specific randomisation. However, the large size of the trial, in which donor blood was sourced from three regional blood transfusion services in two countries and blood was issued based on availability rather than prespecified by request or clinician preference, provides some assurance against potential bias. One of the advantages of this study is that our models were also adjusted for site and other risk factors for each outcome. Moreover, recording of the donor blood haematological characteristics was mostly done by laboratory staff masked to the pack type.

One of the most unexpected findings is that haematological correction was worse in children receiving packed or settled cells than in those receiving whole blood. Indeed, the opposite would be expected given that whole blood is likely to result in more haemodilution in the recipient than packed or settled cells. One obvious explanation is that the haemoglobin concentration of packed or settled cells in a pack was lower than the required range. However, our quality control measures showed that the median and IQR for haemoglobin concentration in packed cells, settled cells, and whole blood were all within the expected range, making this explanation unlikely.^15^ We also weighed each pack and recorded the volume of blood transfused, allowing us to exclude non-adherence to transfusion volume. Therefore, we conclude that guidelines stating that 10 mL/kg of red cell concentrate (as packed or settled cells) is equivalent to 20 mL/kg of whole blood are not correct and should be reviewed. However, given the added expense of preparing components, the revision of guidelines to increase volume of red cell components to the whole blood equivalent would involve a more complex calculation; our data suggest that 30 mL/kg whole blood equates to approximately 20 mL/kg packed or settled cells. Moreover, we have shown that for children with severe anaemia, using red cell concentrates did not show evidence of improved safety in terms of fluid overload complications. At the current recommended volumes, children are exposed to additional transfusions and thus, more donor exposure with the inherent risk of adverse reactions and potential transfusion-transmitted infections. Detection of transfusion-transmitted infections were not included in the study protocol or the extended follow-up, which could have detected transfusion-transmitted infections,^14^ suggesting a possible limitation.

The other major users of blood transfusion services in Africa are the maternity services for emergency management of mothers with bleeding complications. Treatment of anaemia by packed cells does not replace the volume deficit and mothers require additional intravenous fluids to maintain total blood volume. Transfusing whole blood could help to avoid this additional requirement.^23^ Together with the findings from TRACT, our study suggests that component preparation to produce packed or settled cells might not be the optimal strategy for the two largest user groups of transfusion services (children and pregnant women). In Zimbabwe, a transfusion with whole blood is nearly 16% cheaper per pack than using packed cells,^24^ suggesting that transfusion with whole blood could lead to considerable savings. The current process of producing red cell concentrates for paediatric and neonatal transfusion (pedipacks) could be more readily replaced at the point of blood collection from a donor by directly splitting the donation into a number of smaller packs (whole blood pedipacks), which is now a standard practice within the Malawi blood transfusion service.

In conclusion, our study suggests that the use of packed or settled cells rather than whole blood leads to additional transfusions; thus increasing the use of a scarce resource in most of sub-Saharan Africa. Although further consideration should be given by blood transfusion services on how to streamline requirements to include whole blood, as well as when and for whom whole blood transfusion is most needed,^22^ rather than a one-size-fits-all policy for all regional blood transfusion services, a clinical trial comparing whole blood transfusion with red cell concentrates might be needed to inform policy makers.

## Data sharing

The data used in these analyses was collected as part of the TRACT trial, sponsored by Imperial College London, and is stored securely at MRC Clinical Trials Unit at University College London. The datasets analysed in our study are available from the corresponding author upon reasonable request (k.maitland@imperial.ac.uk). We thank all participants and staff from the centres participating in the TRACT trial.

## Declaration of interests

We declare no competing interests.

## Supplementary Material

French translation of the abstract
