# Supplementary material for: Whole blood versus red cell concentrates for children with severe anaemia: a secondary analysis of the Transfusion and Treatment of African Children (TRACT) trial
Source: Lancet Glob Health. 2022 Feb 15;10(3):e360–8. doi: 10.1016/S2214-109X(21)00565-9 (PMC8864302; doi:10.1016/S2214-109X(21)00565-9)
Supplement: French translation of the abstract [file mmc1.pdf]

THE LANCET
Global Health

Supplementary appendix 1

This translation in French was submitted by the authors and we reproduce it as supplied. It has not been peer reviewed. *The Lancet*'s editorial processes have only been applied to the original in English, which should serve as reference for this manuscript.

Cette traduction en français a été proposée par les auteurs et nous l'avons reproduite telle quelle. Elle n'a pas été examinée par des pairs. Les processus éditoriaux du *Lancet* n'ont été appliqués qu'à l'original en anglais et c'est cette version qui doit servir de référence pour ce manuscrit.

Supplement to: George EC, Uyoga S, M'baya B, et al. Whole blood versus red cell concentrates for children with severe anaemia: a secondary analysis of the Transfusion and Treatment of African Children (TRACT) trial. *Lancet Glob Health* 2022; **10:** e360–68.

Sang total contre concentrés de globules rouges pour les enfants atteints d'anémie sévère : une analyse secondaire de l'Essai Transfusion et Traitement des Enfants Africains (TRACT)

Résumé

Contexte : L'essai TRACT a établi le moment de la transfusion chez les enfants atteints d'anémie non compliquée (hémoglobine à 4-6 g/dL) et le volume optimal (20 vs 30 ml/kg de sang total ou 10 vs 15 ml/kg de concentrés de globules rouges) pour la transfusion chez les enfants admis à l'hôpital avec une anémie sévère (hémoglobine < 6 g/dL) au jour 28 ou de la mortalité (critère d'évaluation principal). Les données sur la sécurité des composants sanguins étant rares, nous avons réalisé une analyse secondaire pour examiner la sécurité et efficacité des différents types de poches (sang total vs concentrés de globules rouges) sur les évolutions cliniques.

Méthodes : Cette étude est une analyse secondaire des données de l'essai TRACT limitée aux personnes qui ont reçu une transfusion immédiate (en utilisant du sang total ou des concentrés de globules rouges). TRACT était un essai ouvert, multicentrique, factoriel et randomisé mené dans trois hôpitaux en Ouganda (Soroti, Mbale et Mulago) et un hôpital au Malawi (Blantyre). L'essai a porté sur des enfants âgés de 2 mois à 12 ans admis à l'hôpital pour une anémie sévère (hémoglobine < 6 g/dL). Le type de produit sanguin utilisé (fourni par les banques de sang) était basé uniquement sur la disponibilité à ce moment-là. Les résultats étaient la récupération de l'hémoglobine à 8 heures et à 180 jours, les besoins de re-transfusion, la durée du séjour à l'hôpital, les changements des fréquences cardiaques et respiratoires jusqu'au 180e jour, et le principal critère d'évaluation clinique (mortalité jusqu'au 28e jour et au 180e jour, et réadmission jusqu'au 180e jour), mesurés à l'aide de modèles de régression multivariés.

Résultats : Entre le 17 septembre 2014 et le 15 mai 2017, 3199 enfants atteints d'anémie sévère ont été recrutés dans l'essai TRACT. 3188 enfants ont été pris en compte dans notre analyse secondaire. L'âge médian était de 37 mois (IQR 18-64). Le sang total était le premier conditionnement fourni pour 1632 (41%) des 3992 transfusions. La récupération de l'hémoglobine à 8 heures était significativement plus faible chez les enfants qui avaient reçu des culots globulaires ou des concentrés des globules rouges que chez ceux qui avaient reçu du sang total, avec une moyenne de 1,4 g/dL (IC 95 % -1,6 à -1,1) chez les enfants qui avaient reçu 30 mL/kg et -1,3 g/dL (-1,5 à -1,0) chez ceux qui ont reçu 20 ml/kg de culots globulaires par rapport au sang total, et de -1,5 g/dL (-1,7 à -1,3) chez ceux qui ont reçu 30 ml/kg et de -1,0 g/dL (-1,2 à -0,9) chez ceux qui ont reçu 20 ml/kg de culots globulaires par rapport au sang total (globalement p< 0,0001). Comparé au sang total, les enfants qui ont reçu du sang sous forme de culots globulaires ou de concentrés des globules rouges lors de leur première transfusion avaient plus de chances de recevoir une deuxième transfusion (odds ratio 2,32, IC 95 % 1,30 à 4,12 pour les concentrés des globules rouges et 2,97, 2,18 à 4,05 pour les culots globules rouges; p< 0,001) et des journées d'hospitalisation plus longues (HR 0,94, IC 95 % 0,81 à 1,10

pour les concentrés des globules rouges et 0,86, 0,79 à 0,94 pour les culot globulaires rouges; p= 0,0024). Il n'y avait pas d'association entre le type de sang fourni pour la première transfusion et la mortalité à 28 jours ou 180 jours, ou la réadmission à l'hôpital pour une cause quelconque. 823 (26%) des 3188 enfants ont présenté une tachycardie sévère et 2077 (65%) une tachypnée, mais ces complications se sont résolues avec le temps. Aucun enfant n'a présenté de manifestations de surcharge cardiopulmonaire confirmée.

Interprétation : Notre étude suggère que l'utilisation du concentré des globules rouges ou du culot globulaire plutôt que de sang total conduit à des transfusions supplémentaires, augmentant l'utilisation d'une ressource rare dans la plupart de l'Afrique subsaharienne. Ces résultats ont des implications substantielles en termes de coûts pour la transfusion sanguine et les services de santé. Néanmoins, un essai clinique comparant la transfusion de sang total aux concentrés de globules rouges pourrait être nécessaire pour informer les décideurs politiques.

Source de financement : UK Medical Research Council (MRC) et le Département pour le Développement International.

Droit d’auteur : © 2022 L’(es) Auteur(s). Publié par Elsevier Ltd. Ceci est un article libre

accès sous licence CCBY 4.0.
