# Supplementary material for: Whole blood versus red cell concentrates for children with severe anaemia: a secondary analysis of the Transfusion and Treatment of African Children (TRACT) trial
Source: Lancet Glob Health. 2022 Feb 15;10(3):e360–8. doi: 10.1016/S2214-109X(21)00565-9 (PMC8864302; doi:10.1016/S2214-109X(21)00565-9)
Supplement: Supplementary appendix [file mmc2.pdf]

THE LANCET
Global Health

Supplementary appendix 2

This appendix formed part of the original submission and has been peer reviewed. We post it as supplied by the authors.

Supplement to: George EC, Uyoga S, M'baya B, et al. Whole blood versus red cell concentrates for children with severe anaemia: a secondary analysis of the Transfusion and Treatment of African Children (TRACT) trial. *Lancet Glob Health* 2022; **10:** e360–68.

Appendix: Whole blood versus red cell concentrates for children with severe anaemia: a secondary analysis of the Transfusion and Treatment of African Children (TRACT) trial

TRACT Trial team listed at the end

TRACT Trial design

TRACT was an open-label, multicentre, factorial randomised trial in three hospitals in Uganda (Mulago National Referral Hospital, Mbale and Soroti Regional Referral Hospitals) and one in Malawi (Queen Elizabeth Central Hospital, Blantyre) in children admitted to hospital with severe anaemia (haemoglobin<6g/dl).

Eligibility Criteria

Inclusion Criteria

- Aged 2 months to 12 years
- Severe anaemia (SA) (Hb<6g/dl) within a hospital admission and not previously transfused within this admission: if Hb measurement is >24h post-admission, then child should not have been previously transfused in this admission.
- Carer willing/able to provide consent

Exclusion Criteria

Children with known chronic disease (kidney or liver failure, malignancies, heart failure, congenital heart disease) or admitted for burns, trauma or surgery were excluded, as were children already transfused during the admission and exclusively breast-fed infants(1).

In one stratum, children with complicated severe anaemia (one or more of haemoglobin<4g/dl, reduced consciousness, respiratory distress, acute haemoglobinuria(2) or disclosed sickle cell disease) were randomised 1:1 to 30ml/kg whole-blood (15ml/kg packed-settled cells) vs. 20ml/kg whole-blood (10ml/kg packed-settled cells). In the second stratum, children with uncomplicated severe anaemia (4-6g/dl without severity signs) were randomised 1:1:2 to immediate whole-blood equivalent transfusion of 30ml/kg whole-blood (15ml/kg packed-settled cells) vs 20ml/kg whole-blood (10ml/kg packed-settled cells) vs no immediate transfusion (until or unless severity criteria were met).(3) The trial randomisations examining (a) immediate vs. no immediate transfusion in children with uncomplicated anaemia and (b) volume of transfusions (30mls/kg vs 20 mls/kg) have previously been reported in companion manuscripts(3, 4). All children were also randomised simultaneously to 3 months post-discharge adjunctive micronutrient supplementation vs 3 months of treatment with iron and folate (standard of care), and to 3 months of cotrimoxazole prophylaxis vs none which have been previously reported(5).

Randomisation was stratified by site and other factorial randomisations. Lists were generated using random permuted blocks using variable block sizes and then randomisation envelopes and packs were prepared before the trial. At enrolment sealed consecutively numbered opaque envelopes assigned a TRACT trial number and indicated a clinical pack number. This clinical pack number was within the first 10 packs in the study filing cabinet but not necessarily the first pack. Once opened the pack contained case report forms (CRFs) and a card which simultaneously assigned the transfusion intervention, micronutrient support arm and whether they would receive cotrimoxazole or not.

Transfusions supplies

In Uganda, the supply of donor packs to Mulago National Referral Hospital was from Nakasero National BTS, Kampala. Mbale and Soroti Regional Referral Hospitals received supplies from Mbale Regional BTS. In Malawi, Blantyre Regional BTS supplies blood packs to Queen Elizabeth Central Hospital Blantyre. In Malawi the BTS is an independent trust operating outside of the Ministry of Health owned by the government. In Uganda, the BTSs services are an autonomous service nationally coordinated by the Ministry of Health. For both countries the BTS are responsible for collecting, processing and testing all blood. All the blood packs were screened by the BTS for transfusion transmitted infections (including Human Immunodeficiency Virus, Hepatitis B surface

antigen; Hepatitis C antibodies and Syphilis). As is standard practice in both countries blood transfusions were not leucocyte-reduced during processing(6, 7). From the BTSs the respective hospital blood banks were responsible for collection, transport, storing for blood donations for BTSs, receiving and processing requests for transfusion from the clinical service including cross matching and issuing blood for transfusion. Three pack types produced by local BTSs were supplied for use in the trial(8): whole blood packs that were collected from donors without need for preparation and then stored in two sizes - adult packs or small bags (prepared via closed system of transfer bags; also referred to as pedi-packs); and two types of red cell concentrates. One type of red cell concentrate was 'settled cells' created using gravity (Uganda only) and the other type was packed cells produced by centrifugation followed by the addition of sodium, adenine, glucose and mannitol (SAGM) solution(8).
Blood banks discarded packs that had exceeded their storage length (expired) defined as >35 days for whole blood and >42 days for packed cells and settled cells, and based on the anticoagulant and additive solution used. To quality control the haematological aspects of the blood packs we measured haemoglobin and haematocrit values on all donor blood packs by using an aliquot collected from the blood line prior to the start of each transfusion, as previously reported(4). Donor units of blood were weighed before and after transfusion and administered in gauged burettes to ensure accurate volume(4).

Statistical analysis:
The original sample size for the transfusion volume question was determined that by randomising 2977 children this would provide 80% power to detect a 30% relative reduction in 28-day mortality from 13.7% in the 30ml/kg group to 9.6% in the 20ml/kg group, assuming 6% of participants were lost to follow-up by six months (allowing for different primary endpoint timing in other randomisations) and two-sided alpha=0.013 (four comparisons across randomisations).

The regression models for 28 day and 180 day mortality have been previously published(4). "Baseline predictors of mortality following transfusion were identified using unstratified Cox regression models based on backwards elimination with exit p=0.05 including non-linearity by fractional polynomials where p<0.05 (Stata mfp), forcing the interaction between randomised transfusion strategy and temperature at screening (continuous factor with 5 knot spline as above) into the model."

The competing risks regression models for readmission outcome have been previously published(9) "For the primary outcome, sub-hazard ratios for risk of readmission were estimated using a multivariable competing risks regression model, fitted using backwards stepwise elimination on complete cases for those variables in Supplementary Table 1 with < 5% missing data (including 2690 (69%) of 3894 children discharged alive from their primary admission). Continuous variables were modelled as fractional polynomials to allow for non-linearity using Stata mfp (alpha = 0.05). The model was refitted on cases that were complete on initially selected variables, and then other excluded variables (including those with > 5% missing data) were incorporated one-by-one. All additional factors with p < 0.1 were included in a second backwards elimination, retaining all originally selected factors regardless of significance. Interactions between the final included variables were explored, and backwards elimination performed on all interactions with p < 0.01. Variables with p < 0.05 in the final model were considered significant. For each secondary outcome, a competing risks model included all variables from the all-cause model, then considered the other candidate variables as above. Finally, models containing all factors identified as predictors of malaria or anaemia admissions were fitted together and a test of heterogeneity of effect on these two different causes was performed for each factor using stacked regression.

Heart rate and respiratory rate was compared between groups receiving different blood pack types over time using generalised estimating equations (GEE) (independent correlation structure) adjusting for the fixed independent categorical effects of randomised group, site, and scheduled visit week as well as the continuous fixed covariates of baseline measure and baseline measure by visit interaction. The closest measurement to each scheduled visit date within equally spaced windows was used as the measurement at each scheduled visit. Significance tests were based on least-squares means using a two-sided $\alpha = .05$ (two-sided 95% confidence intervals). Analysis was implemented using Stata version 16.1 (using xtgee).

Supplemental Figure 1: Trial Consort and follow up

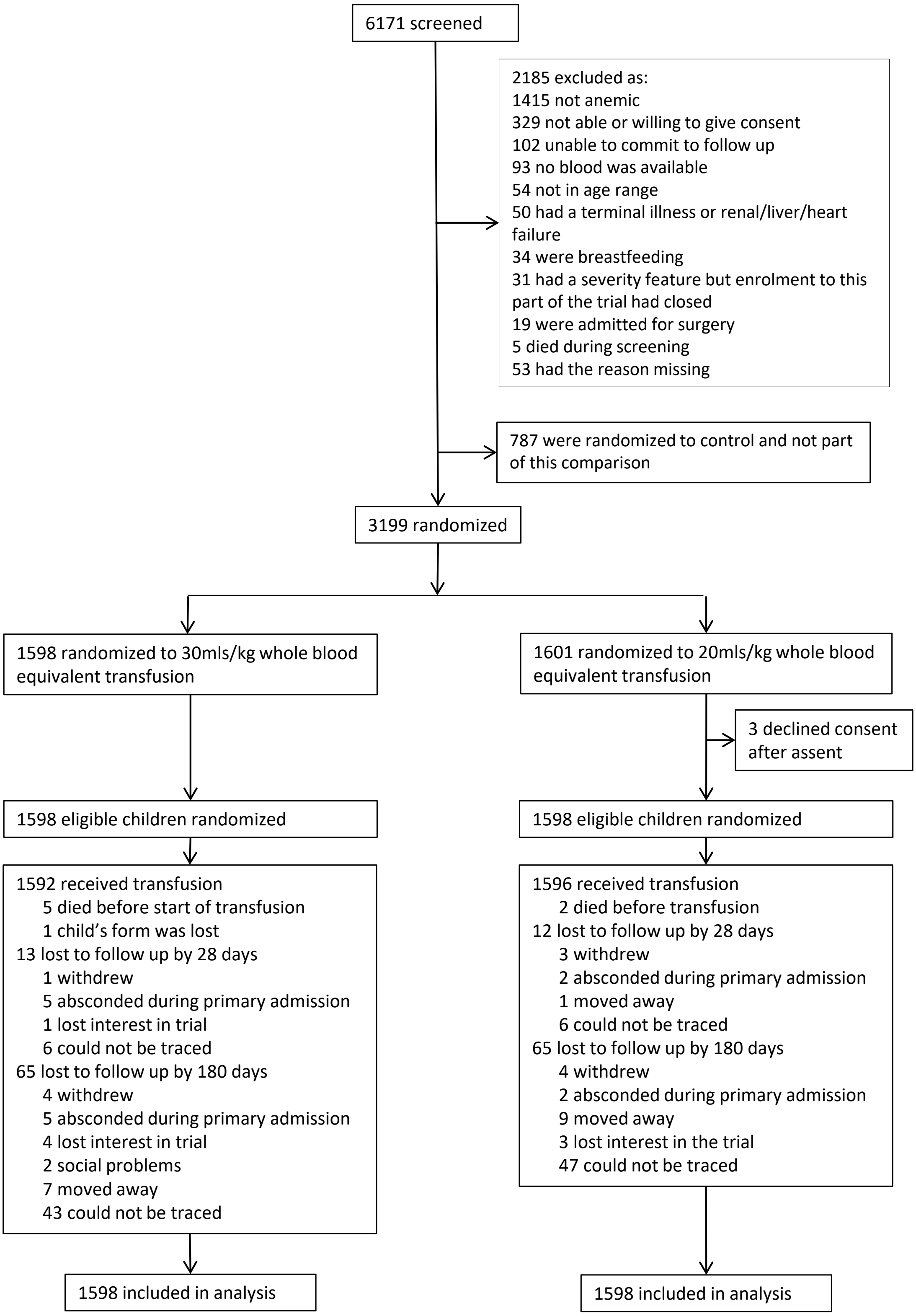

Footnote: This figure is adapted from the original published within the clinical trial report(4)
Of the 3196 children potentially eligible for the secondary analysis 7 children died before receipt of a transfusion (5 in 30 mls/kg arm and 2 in the 20mls/kg arm) and 1 had no form confirming transfusion leaving 3188 children considered in the current analysis.

Supplemental Table 1: SAEs and solicited AEs reported in the TRACT trial in those randomised to an immediate 20ml/kg or 30ml/kg blood transfusion.

	Total<br>N participants<br>(% of 3196)
Any serious adverse event [number of events]	847 (27%) [1152]
Any anaemia SAE [number of events]	454 (14%) [615]
Any malaria SAE [number of events]	237 (7%) [268]
Any sepsis SAE [number of events]	145 (5%) [180]
Any haemoglobinuria SAE [number of events]	96 (3%) [115]
Suspected allergic reactions* **	45 (1%)
Suspected transfusion related lung injury* ‡ **	5 (<1%)
Suspected raised intracranial pressure**	1 (<1%)

‡ suspected pulmonary overload or transfusion-related acute lung injury (TRALI) or transfusion-related cardiac overload (TACO)

** Suspected allergic reaction: 3 grade 4, 12 grade 3, 28 grade 2 and 2 grade 1. Suspected transfusion related lung injury: 2 grade 5 (death): One unlikely related to transfusion and the other unrelated to transfusion, 3 grade 3 (1 probably related to transfusion, 1 unrelated and 1 unlikely related). Suspected raised intracranial pressure (1 grade 3, unrelated to transfusion).

Supplementary Table 2: Numbers of events and unadjusted estimates of effect for blood pack type.

Outcome	Number (%) events in children receiving whole blood packs	Number (%) events in children receiving packed cells packs	Number (%) events in children receiving settled cells packs	Unadjusted estimate (95% CI) (packed cells vs whole blood packs)	Unadjusted estimate (95% CI) (settled cells vs whole blood packs)	Overall Wald p-value
			Time to event analyses (Hazard ratios)			
28 day mortality*	42/1404 (3.0%)	29/692 (4.2%)	49/1092 (4.5%)	1.41 (0.88, 2.27)	1.51 (1.00, 2.28)	0.12
180 day mortality*	105/1404 (7.5%)	67/692 (9.7%)	109/1092 (10.0%)	1.34 (0.99, 1.81)	1.35 (1.03, 1.76)	0.06
Readmissions - all cause§	256/1376 (18.6%)	107/675 (15.9%)	211/1061 (19.9%)	0.87 (0.69, 1.08)	1.09 (0.91, 1.31)	0.14
Readmissions - anaemia§	168/1376 (12.2%)	63/675 (9.3%)	157/1061 (14.4%)	0.77 (0.58)	1.24 (1.00, 1.55)	0.005
Readmissions - DUS§	70/1376 (5.1%)	7/675 (1.0%)	76/1061 (7.2%)	0.21 (0.09, 0.45)	1.43 (1.03, 1.98)	<0.0001
Readmissions - Malaria§	110/1376 (8.0%)	21/675 (3.1%)	76/1061 (7.2%)	0.39 (0.25, 0.62)	0.90 (0.67, 1.20)	0.0004
Time to discharge§	1368/1404 (97.4%)	659/692 (95.2%)	1047/1092 (95.9%)	0.90 (0.83, 0.97)	0.89 (0.84, 0.95)	0.004
			Change in haemoglobin analyses (g/dl)			
8 hour haemoglobin - 20mls/kg¥	-	-	-	-0.83 (-1.01, -0.65)	-0.81 (-0.96, -0.65)	<0.0001
8 hour haemoglobin - 30mls/kg¥	-	-	-	-0.90 (-1.07, -0.72)	-1.33 (-1.48, -1.17)	<0.0001
180 day haemoglobin¥	-	-	-	-0.52 (-0.81, -0.23)	-0.59 (-0.83, -0.35)	<0.0001
			Second transfusions (Odds ratio)			
Odds of second transfusion°	147/1404 (10.5%)	83/692 (12.0%)	266/1092 (24.4%)	1.17 (0.88, 1.55)	2.75 (2.21, 3.43)	<0.0001

Overall Wald test is on 2 degrees of freedom comparing both adjusted estimates to the null.
DUS: Dark Urine Syndrome.
*Cox regression.
§Competing risks regression. For readmission the competing event was death post-discharge, for time to discharge it was death during initial admission.
¥Linear regression.
°Logistic regression.

Supplementary Table 3: Model describing risk factors for mortality at 28 days

Factor at randomisation	Hazard ratio
Pack type: Whole blood	1
Packed cells	0.99 (0.48, 2.04)
Settled cells	1.12 (0.64, 1.95)
Malaria	0.53 (0.35, 0.81)
Oxygen saturation	0.91 (0.88, 0.94)
Respiratory rate	1.03 (1.01, 1.04)
Lactate	1.10 (1.04, 1.16)
Fits	1.86 (1.03, 3.35)
HIV positive	2.84 (1.41, 5.70)
Blantyre Coma Score: 5	1
4	0.81 (0.30, 2.16)
3	2.01 (0.87, 4.63)
2	2.39 (1.16, 4.93)
1	2.84 (0.73, 11.05)
0	2.94 (0.94, 9.23)
Patient blood group: O	1
A	1.03 (0.63, 1.68)
B	0.76 (0.44, 1.31)
AB	2.65 (1.44, 4.90)
Site: Mbale, Uganda	1
Blantyre, Malawi	0.78 (0.35, 1.72)
Mulago, Uganda	1.22 (0.57, 2.64)
Soroti, Uganda	1.09 (0.59, 1.99)

Estimates were also adjusted for randomised comparison, continuous variation in temperature, and the interaction between the two with the use of natural cubic splines.

Supplementary Table 4: Model describing risk factors for mortality at 180 days

Factor at randomisation	Hazard ratio
Pack type: Whole blood	1
Packed cells	1.11 (0.66, 1.85)
Settled cells	1.05 (0.75, 1.46)
Malaria positive	0.44 (0.33, 0.58)
Oxygen saturation (%)	0.94 (0.92, 0.97)
Respiratory rate (brpm)	1.02 (1.01, 1.03)
Lactate (mmol/L)	1.06 (1.02, 1.10)
Fits	0.88 (0.54, 1.42)
HIV positive	2.15 (1.29, 3.59)
Blantyre Coma Score: 5	1
4	0.99 (0.52, 1.89)
3	2.31 (1.30, 4.11)
2	2.13 (1.22, 3.72)
1	4.67 (1.50, 14.52)
0	1.78 (0.59, 5.38)
Patient blood group: O	1
A	1.03 (0.75, 1.41)
B	0.85 (0.61, 1.19)
AB	1.73 (1.10, 2.73)
Site: Mbale, Uganda	1
Blantyre, Malawi	0.51 (0.29, 0.89)
Mulago, Uganda	0.95 (0.56, 1.62)
Soroti, Uganda	1.02 (0.72, 1.46)
Sickle status: AA	1
AS	1.82 (1.08, 3.05)
Unknown SS	0.44 (0.28, 0.69)
Known SS	0.27 (0.15, 0.46)
Blood transfusion ever	1.73 (1.32, 2.28)

Estimates were also adjusted for randomised comparison, continuous variation in temperature, and the interaction between the two with the use of natural cubic splines.

Supplementary Table 5: Risk factors for all cause readmission by 180 days from randomisation

Risk factor at randomisation	Sub Hazard Ratio
Pack type: Whole blood	1
Packed cells	1.05 (0.66, 1.65)
Settled cells	0.85 (0.68, 1.06)
Age in months	0.996 (0.993, 0.999)
Site: Mbale	1
Blantyre	0.71 (0.49, 1.05)
Mulago	0.70 (0.44, 1.11)
Soroti	0.91 (0.72, 1.16)
History of cough	1.10 (0.91, 1.33)
Previous hospital admission	1.51 (1.23, 1.86)
Oral antimalarials	1.01 (0.85, 1.21)
Indrawing	1.51 (1.21, 1.89)
Splenomegaly: Not palpable	1
Enlarged	1.14 (0.95, 1.38)
Gross	1.36 (1.00, 1.86)
HIV positive	2.53 (1.61, 3.97)
Sickle status: AA	1
AS	1.13 (0.68, 1.86)
Unknown SS	0.95 (0.74, 1.22)
Known SS	0.59 (0.44, 0.79)
Age of blood pack	1.01 (1.00, 1.02)
Randomisation after 24 hours of admission	1.27 (0.84, 1.92)
Randomised to 20mls/kg	0.94 (0.79, 1.11)
Missed a dose of medication during follow up	1.45 (1.21, 1.74)
Blood transfusion ever	1.49 (1.11, 2.01)
Malaria positive at primary admission, no previous blood transfusion	0.61 (0.47, 0.81)
Malaria positive at primary admission, previous blood transfusion	1.78 (1.24, 2.54)
Length of stay (per day)	1.01 (0.98, 1.04)
Diarrhoea	0.73 (0.54, 0.99)

Model was built on data from all children in the trial (n=3983) for a separate publication(9) but here is presented in data from 3188 children receiving transfusions in the 30 vs 20mls/kg comparison(4).

Supplementary Table 6: Risk factors for readmission from anaemia by 180 days from randomisation

Risk factor at randomisation	Sub Hazard Ratio
Pack type: Whole blood<br>Packed cells<br>Settled cells	1<br>1.49 (0.87, 2.57)<br>0.90 (0.69, 1.16)
Age in months	0.999 (0.995, 1.002)
Site: Mbale<br>Blantyre<br>Mulago<br>Soroti	1<br>0.59 (0.36, 0.96)<br>0.35 (0.20, 0.63)<br>0.65 (0.48, 0.86)
History of cough	1.05 (0.83, 1.32)
Previous hospital admission	1.74 (1.35, 2.24)
Oral antimalarials	1.13 (0.91, 1.41)
Indrawing	1.19 (0.90, 1.58)
Splenomegaly: Not palpable<br>Enlarged<br>Gross	1<br>1.27 (1.01, 1.61)<br>1.58 (1.10, 2.26)
HIV positive	2.35 (1.38, 3.98)
Sickle status: AA<br>AS<br>Unknown SS<br>Known SS	1<br>1.42 (0.82, 2.48)<br>0.93 (0.68, 1.27)<br>0.40 (0.27, 0.59)
Age of blood pack (days)	1.01 (1.00, 1.03)
Randomisation after 24 hours of admission	1.46 (0.88, 2.40)
Randomised to 20mls/kg	0.90 (0.73, 1.11)
Missed a dose of medication during follow up	1.35 (1.09, 1.68)
Blood transfusion ever	1.75 (1.22, 2.51)
Malaria positive at primary admission, no previous blood transfusion	0.58 (0.40, 0.82)
Malaria positive at primary admission, previous blood transfusion	1.58 (1.02, 2.47)
Length of stay (per day)	1.01 (0.97, 1.05)
Diarrhoea	0.76 (0.52, 1.10)
Haemoglobin at screening	0.85 (0.77, 0.93)
Previous hospital admission for > 24 hours	0.75 (0.54, 1.03)

Model was built on data from all children in the trial (n=3983) for a separate publication(9) but here is presented in data from 3188 children receiving transfusions in the 30 vs 20mls/kg comparison(4). Model includes all risk factors for all cause readmission along with further risk factors for readmission from anaemia.

Supplementary Table 7: Risk factors for readmission from haemoglobinuria by 180 days from randomisation

Risk factor at randomisation	Sub Hazard Ratio
Pack type: Whole blood<br>Packed cells<br>Settled cells	1<br>0.61 (0.12, 3.02)<br>1.08 (0.70, 1.67)
Age in months	1.00 (0.99, 1.01)
Site: Mbale<br>Blantyre<br>Mulago<br>Soroti	1<br>NA (no haemoglobinuria readmissions at site)<br>0.28 (0.06, 1.26)<br>0.96 (0.57, 1.60)
History of cough	1.18 (0.76, 1.83)
Previous hospital admission	2.50 (1.58, 3.98)
Oral antimalarials	1.12 (0.75, 1.66)
Indrawing	1.11 (0.63, 1.96)
Splenomegaly: Not palpable<br>Enlarged<br>Gross	1<br>1.19 (0.72, 1.94)<br>0.73 (0.35, 1.53)
Sickle status: AA<br>AS<br>Unknown SS<br>Known SS	1<br>0.99 (0.33, 2.97)<br>0.06 (0.01, 0.47)<br>0.31 (0.13, 0.73)
Age of blood pack (days)	1.00 (0.98, 1.02)
Randomisation after 24 hours of admission	1.85 (0.79, 4.33)
Uncomplicated severe anaemia (in stratum B of trial)	1.05 (0.59, 1.87)
Missed a dose of medication during follow up	1.58 (1.08, 2.32)
Blood transfusion ever	2.91 (0.90, 9.38)
Malaria positive at primary admission, no previous blood transfusion	1.72 (0.60, 4.95)
Malaria positive at primary admission, previous blood transfusion	0.87 (0.26, 2.88)
Length of stay (per day)	1.01 (0.93, 1.09)
Diarrhoea	0.65 (0.31, 1.40)
Hemoglobinuria	1.88 (1.14, 3.09)
Culture positive	3.67 (1.46, 9.18)
Liver >2cm below costal margin	1.33 (0.82, 2.17)
Granulocytes (per 10 x 10^9/L) on primary admission	1.03 (1.01, 1.05)

Model was built on data from all children in the trial (n=3983) for a separate publication(9) but here is presented in data from 3188 children receiving transfusions in the 30 vs 20mls/kg comparison. Model includes all risk factors for all cause readmission along with further risk factors for readmission from haemoglobinuria.

Supplementary Table 8: Risk factors for readmission from malaria by 180 days from randomisation

Risk factor at randomisation	Sub Hazard Ratio
Pack type: Whole blood<br>Packed cells<br>Settled cells	1<br>0.59 (0.26, 1.39)<br>0.70 (0.47, 1.02)
Age in months	0.99 (0.98, 1.00)
Site: Mbale<br>Blantyre<br>Mulago<br>Soroti	1<br>0.99 (0.48, 2.06)<br>1.07 (0.46, 2.49)<br>1.11 (0.72, 1.72)
History of cough	1.16 (0.82, 1.65)
Previous hospital admission	1.18 (0.81, 1.73)
Oral antimalarials	0.77 (0.55, 1.06)
Indrawing	1.36 (0.89, 2.08)
Splenomegaly: Not palpable<br>Enlarged<br>Gross	1<br>1.07 (0.76, 1.51)<br>0.99 (0.52, 1.86)
HIV positive	1.01 (0.31, 3.34)
Sickle status: AA<br>AS<br>Unknown SS<br>Known SS	1<br>0.68 (0.21, 2.23)<br>1.07 (0.57, 1.98)<br>0.43 (0.20, 0.90)
Age of blood pack (days)	1.01 (0.99, 1.04)
Randomisation after 24 hours of admission	1.16 (0.54, 2.46)
Randomised to 20mls/kg arm	0.82 (0.60, 1.12)
Missed a dose of medication during follow up	1.30 (0.93, 1.82)
Blood transfusion ever	2.78 (1.32, 5.86)
Malaria positive at primary admission, no previous blood transfusion	1.80 (0.88, 3.67)
Malaria positive at primary admission, previous blood transfusion	0.67 (0.30, 1.51)
Temperature gradient	1.94 (1.17, 3.20)
Randomised to receive cotrimoxazole	0.76 (0.55, 1.04)
Walk unaided prior to illness	2.05 (1.01, 4.18)
Type of homestead: Urban<br>Semi urban<br>Rural	1<br>0.63 (0.17, 2.32)<br>2.89 (1.13, 7.43)
Platelets (per 100 x 10^9/L) at admission	1.00 (0.99, 1.001)
Length of stay (per day)	1.01 (0.95, 1.08)
Monocytes (per 10^9/L) at admission	0.86 (0.74, 1.00)
Diarrhea	0.94 (0.52, 1.66)

Model was built on data from all children in the trial (n=3983) for a separate publication(9) but here is presented in data from 3188 children receiving transfusions in the 30 vs 20mls/kg comparison. Model includes all risk factors for all cause readmission along with further risk factors for readmission from malaria.

Supplementary Table 9: Risk factors for time to discharge from randomisation

Risk factor at randomisation	
Pack type: Whole blood<br>Packed cells<br>Settled cells	1<br>0.94 (0.81, 1.10)<br>0.86 (0.79, 0.94)
Site Mbale<br>Blantyre<br>Mulago<br>Soroti	1<br>1.07 (0.92, 1.25)<br>0.69 (0.58, 0.82)<br>0.55 (0.50, 0.61)
MUAC z-score	1.07 (1.04, 1.11)
Sickle status – AA<br>AS<br>unknown SS<br>Known SS	1<br>0.85 (0.67, 1.08)<br>1.03 (0.94, 1.12)<br>1.02 (0.93, 1.13)
Homestead – Urban<br>Semi-urban<br>Rural	1<br>0.93 (0.81, 1.06)<br>0.87 (0.77, 0.99)
Respiratory distress	0.84 (0.77, 0.92)
Impaired consciousness	0.78 (0.71, 0.86)
Oxygen saturation (%)	1.03 (1.02, 1.04)
History of fever for more than 14 days	0.75 (0.64, 0.88)
Fits on admission	0.85 (0.74, 0.97)
Crackles	0.81 (0.71, 0.92)
Sunken eyes	0.84 (0.70, 0.98)
Cold hands	0.83 (0.70, 0.98)
Liver >2cm below costal margin	0.92 (0.85, 1.00)
Diarrhoea	0.87 (0.78, 0.96)
Jaundice	0.91 (0.85, 0.98)
Admission into another hospital >24 hours	0.84 (0.76, 0.93)
Received oral traditional medicine in this illness	0.91 (0.82, 1.00)
Received antibiotics on admission	0.87 (0.80, 0.94)
Pack age (days)	0.994 (0.990, 0.998)
Time of admission	0.93 (0.86, 1.00)
Blood culture positive	0.75 (0.62, 0.91)
HIV positive	0.76 (0.60, 0.95)

Supplementary Table 10: Risk factors for haemoglobin level at 8 hours from randomisation

Risk factor at randomisation	Mean difference (95% CI)
Pack type Whole blood – 20mls/kg<br>Packed cells – 20mls/kg<br>Settled cells - 20mls/kg	0<br>-1.28 (-1.52, -1.04)<br>-1.07 (-1.23, -0.92)
Pack type Whole blood – 30mls/kg<br>Packed cells – 30mls/kg<br>Settled cells – 30mls/kg	0<br>-1.36 (-1.59, -1.13)<br>-1.50 (-1.65, -1.34)
Haemoglobin at screening	0.88 (0.80, 0.96)
Site: Mbale<br>Blantyre<br>Mulago<br>Soroti	0<br>0.03 (-0.18, 0.22)<br>-0.01 (-0.21, 0.20)<br>0.36 (0.23, 0.49)
Log(age (months))	-0.45 (-0.50, -0.39)
Pack age (days)	-0.01 (-0.02, -0.005)
$(\text{Pack haemoglobin})^{0.5}$ (g/dL)	0.56 (0.49, 0.63)
Log(Heart rate (bpm))	-0.17 (-0.25, -0.08)
Malaria positive	-0.08 (-0.18, 0.01)
Splenomegaly – Not palpable<br>Enlarged<br>Gross	0<br>-0.14 (-0.24, -0.04)<br>-0.25 (-0.43, -0.07)
Previous blood transfusion	-0.10 (-0.20, 0.004)
Haemoglobin <4g/dl at screening	0.21 (0.04, 0.38)
Kwashiorkor	0.29 (-.005, 0.59)
$(\text{MCHC})^3$ (g/dL)	-0.04 (-0.08, -0.005)
$(\text{MCV})^{-2}$ (fL)	0.00006 (0.00002, 0.0001)
Oral antimalarials prior to admission	-0.10 (-0.19, -0.005)

Log=natural logarithm. MCHC: mean corpuscular haemoglobin concentration. MCV: mean corpuscular volume.

Supplementary Table 11: Risk factors for haemoglobin level at 180 days from randomisation

Risk factor at randomisation	Mean difference (95% CI)
Pack type Whole blood	0
Packed cells	0.02 (-0.35, 0.40)
Settled cells	0.12 (-0.10, 0.33)
Randomised to 30mls/kg	-0.12 (-0.29, 0.05)
Haemoglobin at screening	0.11 (0.03, 0.20)
Site Mbale	0
Blantyre	0.61 (0.26, 0.97)
Mulago	0.03 (-0.37, 0.43)
Soroti	0.78 (0.54, 1.02)
Age (months)	0.09 (0.06, 0.13)
Previous blood transfusion	-0.24 (-0.45, -0.02)
Previous hospital admission	-0.43 (-0.66, -0.20)
Previous hospital admission > 24 hours	0.29 (0.04, 0.55)
Haemoglobinuria	0.38 (0.14, 0.61)
Fits at admission	0.53 (0.18, 0.87)
Homestead – Urban	0
Semi-urban	0.09 (-0.28, 0.46)
Rural	-0.34 (-0.66, -0.03)
Sickle status AA	0
AS	-0.61 (-1.15, -0.07)
Unknown SS	-3.53 (-3.79, -3.27)
Known SS	-3.93 (-4.21, -3.66)
Walk unaided	0.25 (-0.02, 0.52)
(Pack haemoglobin)$^{-2}$ (g/dL)	0.00017 (0.000029, 0.00021)

Supplementary Table 12: Risk factors for a second transfusion during admission.

Risk factor at randomisation	Mean difference (95% CI)
Pack type Whole blood<br>Packed cells<br>Settled cells	1<br>2.32 (1.30, 4.12)<br>2.97 (2.18, 4.05)
Randomised to 20mls/kg	0.49 (0.39, 0.62)
Haemoglobinuria	1.83 (1.37, 2.44)
Blood transfusion ever	1.64 (1.27, 2.13)
Respiratory distress	1.44 (1.06, 1.97)
Impaired consciousness	1.51 (1.10, 2.06)
Age (months)	1.01 (1.00, 1.02)
Haemoglobin at screening	0.50 (0.44, 0.56)
Oxygen saturation (%)	0.97 (0.94, 1.00)
Height/Length z-score	1.14 (1.04, 1.23)
MUAC z-score	0.93 (0.92, 1.05)
Pack age (days)	1.04 (1.02, 1.05)
Pack haemoglobin (g/dl)	0.88 (0.84, 0.92)
Site – Mbale, Uganda<br>Blantyre, Malawi<br>Mulago, Uganda<br>Soroti, Uganda	1<br>0.17 (0.08, 0.35)<br>0.85 (0.49, 1.49)<br>0.66 (0.48, 0.92)
Splenomegaly – Not palpable<br>Enlarged<br>Gross	1<br>1.05 (0.81, 1.38)<br>2.04 (1.39, 3.01)
Homestead – Urban<br>Semi-urban<br>Rural	1<br>1.82 (0.97, 3.43)<br>1.85 (1.06, 3.22)
Patient blood group – A<br>B<br>AB<br>O	1<br>1.26 (0.92, 1.73)<br>0.82 (0.47, 1.43)<br>0.93 (0.69, 1.25)
Sickle Status – AA<br>AS<br>SS unknown<br>SS known	1<br>0.65 (0.31, 1.37)<br>0.47 (0.31, 0.71)<br>1.03 (0.72, 1.47)
Jaundice	1.36 (1.04, 1.77)
MCH (picograms)	1.05 (1.02, 1.08)

MUAC: Mid-upper arm circumference. MCH: mean corpuscular haemoglobin.

Supplementary Table 13: Sensitivity analyses restricting models to specific sites given distribution of blood packs described in Table 1.

Outcomes	Mulago, Uganda and Blantyre, Malawi (N=2048) Packed cells vs whole blood	Soroti and Mbale, Uganda (N=1067) Settled cells vs whole blood
28 day mortality*	1.11 (0.31, 4.04)	0.89 (0.38, 2.10)
180 day mortality*	1.31 (0.45, 3.85)	0.87 (0.54, 1.41)
Readmissions - all cause§	0.90 (0.51, 1.59)	0.87 (0.70, 1.09)
Readmissions - anaemia§	1.25 (0.60, 2.61)	0.90 (0.69, 1.17)
Readmissions - DUS§	Not estimated as too few events	1.05 (0.67, 1.64)
Readmissions - Malaria§	0.98 (0.26, 3.74)	0.61 (0.41, 0.91)
Time to discharge§	1.01 (0.86, 1.19)	0.82 (0.75, 0.90)
8 hour haemoglobin - 20mls/kg¥	-1.05 (-1.40, -0.71)	-1.36 (-1.70, -1.03)
8 hour haemoglobin - 30mls/kg¥	-1.04 (-1.20, -0.88)	-1.41 (-1.58, -1.25)
180 day haemoglobin¥	0.02 (-0.35, 0.39)	0.09 (-0.16, 0.33)
Odds of second transfusion°	1.94 (0.80, 4.71)	3.17 (2.28, 4.40)

All models were adjusted for site. DUS: Dark Urine Syndrome
*Cox regression models (see supplementary material).
§Competing risk models. For readmission the competing event was death post-discharge, for time to discharge it was death during initial admission (see supplementary material).
¥Linear regression models (see supplementary material).
°Logistic regression model (see supplementary material).

Supplementary Table 14a: Sensitivity analyses: Impact of blood pack type on clinical outcomes by subgroups of children with or without malaria.

	Children with malaria		Children with no malaria			
**Outcome**	**Adjusted estimate (95% CI) (packed cells vs whole blood packs)**	**Adjusted estimate (95% CI) (settled cells vs whole blood packs)**	**Adjusted estimate (95% CI) (packed cells vs whole blood packs)**	**Adjusted estimate (95% CI) (settled cells vs whole blood packs)**	**p-value for heterogeneity (packed cells)**	**p-value for heterogeneity (settled cells)**
Time to event analyses (Hazard ratios)						
28 day mortality*	1.05 (0.43, 2.55)	0.94 (0.47, 1.85)	1.03 (0.43, 2.48)	1.42 (0.66, 3.07)	0.97	0.38
180 day mortality*	1.26 (0.67, 2.37)	1.15 (0.76, 1.73)	0.99 (0.55, 1.79)	0.92 (0.57,1.48)	0.48	0.46
Readmissions - all cause§	0.97 (0.58, 1.61)	0.84 (0.65, 1.08)	1.14 (0.68, 1.95)	0.87 (0.62, 1.23)	0.51	0.84
Readmissions - anaemia§	1.25 (0.66, 2.36)	0.84 (0.62, 1.13)	1.81 (0.97, 3.39)	1.02 (0.68, 1.53)	0.25	0.43
Readmissions - DUS§	0.65 (0.13, 3.17)	0.91 (0.58, 1.45)	0.69 (0.04, 11.50)	2.47 (0.82, 7.45)	0.96	0.09
Readmissions - Malaria§	0.47 (0.12, 1.77)	0.54 (0.25, 1.19)	0.66 (0.28, 1.51)	0.74 (0.48, 1.12)	0.58	0.48
Time to discharge§	0.99 (0.84, 1.17)	0.85 (0.77, 0.94)	0.89 (0.74, 1.07)	0.88 (0.77, 1.01)	0.20	0.64
Change in haemoglobin analyses (g/dl)						
8 hour haemoglobin - 20mls/kg¥	-1.13 (-1.41, -0.85)	-1.06 (-1.24, -0.87)	-1.49 (-1.82, -1.18)	-1.09 (-1.36, -0.83)	0.04	0.82
8 hour haemoglobin - 30mls/kg¥	-2.24 (-2.94, -1.54)	-1.19 (-1.81, -0.57)	-1.14 (-1.45, -0.83)	-1.45 (-1.72, -1.19)	0.004	0.66
180 day haemoglobin¥	0.29 (-0.13, 0.72)	0.13 (-0.13, 0.38)	-0.34 (-0.82, 0.13)	0.08 (-0.27, 0.44)	0.009	0.84
Second transfusions (Odds ratio)						
Odds of second transfusion°	1.79 (0.94, 3.41)	2.68 (1.89, 3.80)	3.53 (1.70, 7.35)	3.72 (2.20, 6.30)	0.07	0.27

All models were adjusted for site. DUS: Dark Urine Syndrome.
The p-values from the heterogeneity tests (Wald tests on 1 degrees of freedom) from twenty-two tests were then ranked and compared with critical values from the Benjamini-Hochberg procedure, to account for the multiple testing. All were found to be greater than their corresponding critical values. The most significant heterogeneity tests either indicated quantitative interactions (ie slightly smaller or larger differences in the same direction, 8h haemoglobin) or effects that were small in magnitude without any evidence that each differed from no effect (180 day haemoglobin).
*Cox regression models (see supplementary material).
§Competing risk models. For readmission the competing event was death post-discharge, for time to discharge it was death during initial admission (see supplementary material).
¥Linear regression models (see supplementary material).
°Logistic regression model (see supplementary material).

Supplementary Table 14b: Sensitivity analyses: Impact of blood pack type on selected clinical outcomes by subgroups of children with or without HIV.

	Children with HIV		Children without HIV			
**Outcome**	**Adjusted estimate (95% CI) (packed cells vs whole blood packs)**	**Adjusted estimate (95% CI) (settled cells vs whole blood packs)**	**Adjusted estimate (95% CI) (packed cells vs whole blood packs)**	**Adjusted estimate (95% CI) (settled cells vs whole blood packs)**	**p-value for heterogeneity (packed cells)**	**p-value for heterogeneity (settled cells)**
Time to event analyses (Hazard ratios)						
Time to discharge§	0.94 (0.56, 1.60)	0.84 (0.52, 1.35)	0.94 (0.81, 1.10)	0.86 (0.79, 0.94)	0.99	0.91
Change in haemoglobin analyses (g/dl)						
8 hour haemoglobin - 20mls/kg¥	-0.86 (-1.75, 0.03)	-1.11 (-2.15, -0.10)	-1.28 (-1.55, -1.03)	-1.05 (-1.21, -0.89)	0.37	0.91
8 hour haemoglobin - 30mls/kg¥	-2.50 (-3.81, -1.20)	-1.92 (-3.52, -0.33)	-1.33 (-1.58, -1.09)	-1.48 (-1.64, -1.31)	0.06	0.28
180 day haemoglobin¥	-0.47 (-1.73, 0.59)	0.40 (-1.16, 1.96)	-0.005 (-0.40, 0.39)	0.10 (-0.12, 0.33)	0.35	0.71
Second transfusions (odds ratio)						
Odds of second transfusion°	0.40 (0.06, 2.71)	2.66 (0.48, 14.90	2.63 (1.44, 4.82)	2.95 (2.14, 4.08)	0.06	0.91

Other clinical outcomes (mortality, readmissions and second transfusions) could not be assessed due to the small size of the HIV subgroup (n=98).

All models were adjusted for site. The p-values from the heterogeneity tests (Wald tests on 1 degrees of freedom) from ten tests were then ranked and compared with critical values from the Benjamini-Hochberg procedure, to account for the multiple testing. All were found to be greater than their corresponding critical values.

*Cox regression models (see supplementary material).

§Competing risk models. For time to discharge the competing risk was death during initial admission (see supplementary material).

¥Linear regression models (see supplementary material).

°Logistic regression model (see supplementary material).

Supplementary Figure 2: Cumulative incidence plots by blood pack type for a) all cause readmissions b) anaemia readmissions c) haemoglobinurea readmissions d) malaria readmissions e) time to discharge.

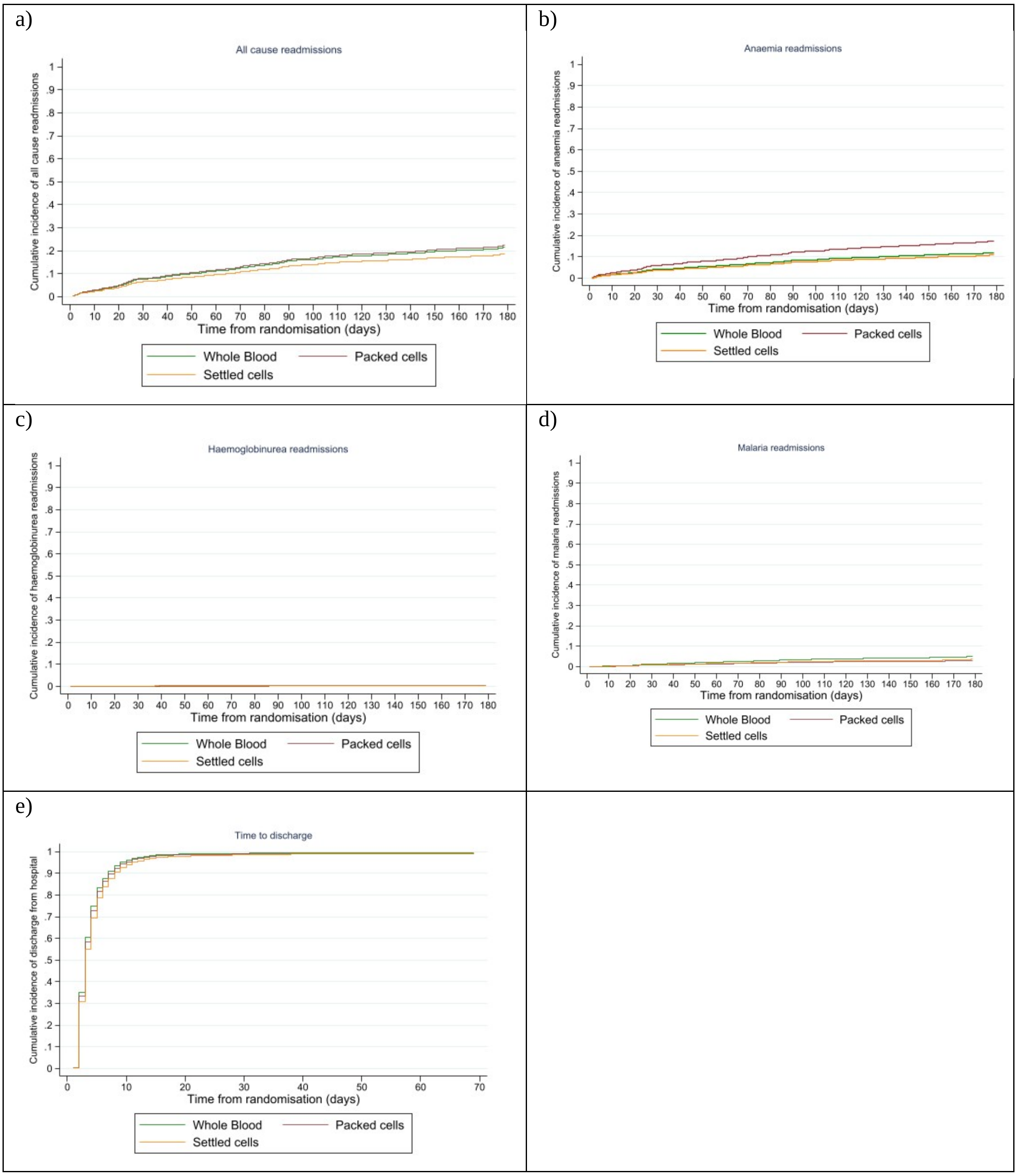

For readmission the competing event was death post-discharge, for time to discharge it was death during initial admission (see supplementary material).

Supplementary Table 15a): Heart rate over time from beginning of first transfusion

		Mean (95% CI) heart rate (beats per min)		
Time point	Randomised arm	Whole blood	Packed cells	Settled cells
Admission	30mls/kg	145.7 (144.1, 147.2)	145.1 (142.7, 147.5)	147.5 (145.6, 149.5)
Admission	20mls/kg	145.3 (143.7, 147.0)	144.3 (141.8, 146.8)	148.0 (146.1, 150.0)
30 minutes	30mls/kg	143.3 (141.8, 144.8)	144.4 (142.1, 146.7)	145.5 (143.6, 147.5)
30 minutes	20mls/kg	143.3 (141.7, 144.8)	142.8 (140.5, 145.1)	147.3 (145.4, 149.2)
60 minutes	30mls/kg	142.6 (141.1, 144.0)	143.3 (141.0, 145.6)	144.3 (142.4, 146.2)
60 minutes	20mls/kg	141.9 (140.4, 143.4)	141.5 (139.4, 143.7)	146.1 (144.3, 147.9)
90 minutes	30mls/kg	140.4 (138.9, 141.9)	141.2 (139.0, 143.5)	142.1 (140.2, 144.0)
90 minutes	20mls/kg	140.5 (139.0, 142.0)	139.8 (137.7, 141.8)	143.9 (142.1, 145.7)
2 hours	30mls/kg	138.6 (137.1, 140.0)	139.7 (137.6, 141.8)	139.0 (137.2, 140.8)
2 hours	20mls/kg	138.9 (137.4, 140.4)	137.7 (135.8, 139.5)	141.0 (139.3, 142.8)
4 hours	30mls/kg	133.4 (131.9, 134.8)	132.5 (130.7, 134.4)	132.8 (130.9, 134.7)
4 hours	20mls/kg	133.8 (132.3, 135.4)	132.2 (130.4, 134.1)	136.0 (134.3, 137.7)
8 hours	30mls/kg	125.3 (123.9, 126.8)	127.3 (125.3, 129.2)	126.6 (124.8, 128.3)
8 hours	20mls/kg	129.6 (126.7, 132.5)	130.0 (128.2, 131.9)	130.1 (128.4, 131.9)
16 hours	30mls/kg	119.0 (117.6, 120.3)	124.1 (122.3, 125.8)	123.0 (121.3, 124.8)
16 hours	20mls/kg	121.9 (120.5, 123.3)	126.2 (124.4, 128.0)	126.4 (124.8, 128.1)
24 hours	30mls/kg	116.5 (115.2, 117.9)	121.3 (119.5, 123.1)	122.8 (121.1, 124.5)
24 hours	20mls/kg	121.2 (119.8, 122.6)	124.0 (122.2, 125.8)	126.1 (124.4, 127.8)
48 hours	30mls/kg	112.9 (111.6, 114.3)	117.2 (115.4, 119.0)	118.5 (116.9, 120.1)
48 hours	20mls/kg	117.0 (115.7, 118.3)	122.6 (120.8, 124.3)	122.1 (120.5, 123.7)

Supplementary Table 15b): Respiratory rate over time from beginning of first transfusion

		Mean respiratory rate (95%CI) (breaths per min)		
Time point	Randomised arm	Whole blood	Packed cells	Settled cells
Admission	30mls/kg	42.4 (41.5, 43.2)	45.2 (43.8, 46.5)	43.5 (42.4, 44.7)
Admission	20mls/kg	42.5 (41.5, 43.5)	44.7 (43.3, 46.1)	44.1 (43.0, 45.3)
30 minutes	30mls/kg	41.9 (41.1, 42.7)	43.9 (42.7, 45.2)	43.0 (41.9, 44.1)
30 minutes	20mls/kg	41.2 (40.4, 42.0)	44.4 (43.1, 45.6)	43.2 (42.1, 44.2)
60 minutes	30mls/kg	41.2 (40.4, 42.0)	43.4 (42.2, 44.6)	42.4 (41.4, 43.5)
60 minutes	20mls/kg	40.9 (40.2, 41.7)	43.8 (42.6, 45.0)	42.3 (41.3, 43.4)
90 minutes	30mls/kg	40.7 (39.9, 41.5)	42.4 (41.2, 43.5)	41.6 (40.6, 42.6)
90 minutes	20mls/kg	40.2 (39.4, 41.0)	42.9 (41.6, 44.1)	41.4 (40.5, 42.4)
2 hours	30mls/kg	39.9 (39.2, 40.7)	40.9 (39.8, 42.0)	40.3 (39.3, 41.3)
2 hours	20mls/kg	39.8 (39.0, 40.5)	41.3 (40.1, 42.5)	40.3 (39.4, 41.3)
4 hours	30mls/kg	38.1 (37.3, 38.8)	38.8 (37.8, 39.8)	38.7 (37.7, 39.6)
4 hours	20mls/kg	38.0 (37.2, 38.8)	38.9 (38.0, 39.8)	38.9 (38.0, 39.8)
8 hours	30mls/kg	36.8 (36.1, 37.5)	37.1 (36.2, 38.0)	36.1 (35.3, 36.9)
8 hours	20mls/kg	36.7 (36.0, 37.5)	37.7 (36.8, 38.7)	36.5 (35.7, 37.3)
16 hours	30mls/kg	34.1 (33.5, 34.7)	34.9 (34.0, 35.7)	33.7 (33.0, 34.5)
16 hours	20mls/kg	34.1 (33.4, 34.7)	36.4 (35.4, 37.3)	34.5 (33.8, 35.2)
24 hours	30mls/kg	33.2 (32.7, 33.8)	34.6 (33.8, 35.5)	33.0 (32.3, 33.6)
24 hours	20mls/kg	33.3 (32.7, 33.9)	35.8 (34.8, 36.7)	33.5 (32.9, 34.2)
48 hours	30mls/kg	31.8 (31.4, 32.3)	33.8 (33.0, 34.6)	31.8 (31.2, 32.4)
48 hours	20mls/kg	32.0 (31.5, 32.5)	34.5 (33.7, 35.3)	32.3 (31.6, 33.0)

References

1. Mpoya A, Kiguli S, Olupot-Olupot P, Opoka RO, Engoru C, Mallewa M, et al. Transfusion and Treatment of severe anaemia in African children (TRACT): a study protocol for a randomised controlled trial. Trials. 2015;16(1):593.
2. Olupot-Olupot P, Engoru C, Uyoga S, Muhindo R, Macharia A, Kiguli S, et al. High Frequency of Blackwater Fever Among Children Presenting to Hospital With Severe Febrile Illnesses in Eastern Uganda. Clinical infectious diseases : an official publication of the Infectious Diseases Society of America. 2017;64(7):939-46.
3. Maitland K, Kiguli S, Olupot-Olupot P, Engoru C, Mallewa M, Saramago Goncalves P, et al. Immediate Transfusion in African Children with Uncomplicated Severe Anemia. The New England journal of medicine. 2019;381(5):407-19.
4. Maitland K, Olupot-Olupot P, Kiguli S, Chagaluka G, Alaroker F, Opoka RO, et al. Transfusion Volume for Children with Severe Anemia in Africa. The New England journal of medicine. 2019;381(5):420-31.
5. Maitland K, Olupot-Olupot P, Kiguli S, Chagaluka G, Alaroker F, Opoka RO, et al. Co-trimoxazole or multivitamin multimineral supplement for post-discharge outcomes after severe anaemia in African children: a randomised controlled trial. Lancet Glob Health. 2019;7(10):e1435-e47.
6. Global status report on blood safety and availability 2016. Geneva: World Health Organization: World Health Organization; 2017. Report No.: 978-92-4-156543-1.
7. Ala F, Allain JP, Bates I, Boukef K, Boulton F, Brandful J, et al. External financial aid to blood transfusion services in sub-Saharan Africa: a need for reflection. PLoS medicine. 2012;9(9):e1001309.
8. Uyoga S, Mpoya A, Olupot-Olupot P, Kiguli S, Opoka RO, Engoru C, et al. Haematological quality and age of donor blood issued for paediatric transfusion to four hospitals in sub-Saharan Africa. Vox Sang. 2019;114(4):340-8.
9. Connon R, George EC, Olupot-Olupot P, Kiguli S, Chagaluka G, Alaroker F, et al. Incidence and predictors of hospital readmission in children presenting with severe anaemia in Uganda and Malawi: a secondary analysis of TRACT trial data. BMC Public Health. 2021;21(1):1480.

Trial Team listing for PubMed

Department of Paediatrics, Mulago Hospital, Makerere University, Kampala, Uganda: S Kiguli, R O Opoka, E Nabawanuka, J Kayaga, C Williams Musika, E Kadama, I Mbwali, L Nuwabaine, R Nakikwaku, J Nsubuga, K Mpande, R Adoo, O Ouma, N K Adia.

Mbale Regional Referral Hospital Mbale, Uganda: P Olupot-Olupot, J Nteziyaremye, C Namanyanga, G Passi, T, Sennyondo, R Adong, CB Okalebo, E Atimango, S Mwamula, J Kapsindet, G Kiluli R Muhindo, G Masifa N Thembo, G Odong.

Soroti Regional Referral Hospital Mbale, Uganda: C Engoru, F Aloroker, M Nakuya, D Aromut M Ariima, M Itipe, MG Atim, M Abeno, B Amede, M Olupot, S Okwi, MG Kulume, G Among, P Onyas, ED Achipa

KEMRI Wellcome Trust Research Programme, Kilifi, Kenya (coordinating centre for the trial and genetics group): K Maitland, A Mpoya, P Maitha, S Uyoga, TN Williams, A Macharia

College of Medicine and Malawi-Liverpool-Wellcome Trust Clinical Research Programme, College of Medicine, Blantyre, Malawi: M Mallewa, G Chagaluka, Y Chimalizeni, N Kennedy, F Kumwenda, E Nkosi, T Sochera, A Malenga, B Gushu, T Phiri, A Chisale, N Mitole, E Chokani, A Munthali

Imperial College London (Trial Sponsor): K Maitland, TN Williams; Nutritional studies: G Frost, K Walsh

MRC Clinical Trials Unit at UCL, London, UK (oversight): DM Gibb, EC George, M Thomason, D Baptiste, L McCabe, AS Walker

Data Management Systems: A. Ali, K Khamis (Kenya) M Madula, G Abongo (Uganda)

Liverpool School of Tropical Medicine, Liverpool, UK: R Heydermann, I Bates, B Urban

Emma Children's Hospital AMC Amsterdam, The Netherlands: M Boele von Hensbroek

Initial and Surnames

K	Maitland
P	Olupot-Olupot
S	Kiguli
G	Chagaluka
F	Alaroker
RO	Opoka
A	Mpoya
K	Walsh
C	Engoru
J	Nteziyaremya
M	Mallewa
N	Kennedy
M	Nakuya
C	Namayanja
J	Kayaga
E	Nabawanuka
T	Sennyondo
D	Aromut
F	Kumwenda
C	Williams Musika
MJ	Thomason
I	Bates
M	Boele von Hensbroek
J	Evans
S	Uyoga
TN	Williams

G	Frost
DM	Gibb
EC	George
AS	Walker
R	Muhindo
G	Odong
G	Masifa
Y	Chimalizeni
L	McCabe
D	Baptiste
E	Kadama
I	Mbwali
L	Nuwabaine
R	Nakikwaku
J	Nsubuga
K	Mpande
R	Adoo
O	Ouma
NK	Adia
R	Adong
CB	Okalebo
E	Atimango
S	Mwamula
J	Kapsindet
G	Kiluli
R	Muhindo
G	Masifa
N	Thembo
G	Odong
M	Ariima
MG	Itipe
M	Abeno
B	Amede
M	Olupot
S	Okwi
MG	Kulume
G	Among
P	Onyas
ED	Achipa
P	Maitha
A	Macharia
E	Nkosi
T	Sochera
A	Malenga
B	Gushu
T	Phiri
A	Chisale
N	Mitole
E	Chokani
A	Munthali
A	Ali
K	Khamis
M	Madula
G	Abongo
R	Heydermann
B	Urban
